# Supplementary material for: Protein Set Transformer: a protein-based genome language model to power high-diversity viromics
Source: Nat Commun. 2025 Nov 23;16:11123. doi: 10.1038/s41467-025-66049-4 (PMC12705698; doi:10.1038/s41467-025-66049-4)
Supplement: Supplementary file 1 — Supplementary Information [file 41467_2025_66049_MOESM1_ESM.pdf]

Supplementary Information

Protein Set Transformer: A protein-based genome language model to power high diversity viromics

Cody Martin[1,2], Anthony Gitter[3,4,5], and Karthik Anantharaman[1,6, 7,*]

[1] Department of Bacteriology, University of Wisconsin-Madison, Madison, WI, USA
[2] Microbiology Doctoral Training Program, University of Wisconsin-Madison, Madison, WI, USA
[3] Department of Biostatistics and Medical Informatics, University of Wisconsin-Madison, Madison, WI, USA
[4] Morgridge Institute for Research, Madison, WI, USA
[5] Department of Computer Sciences, University of Wisconsin-Madison, Madison, WI, USA
[6] Department of Integrative Biology, University of Wisconsin-Madison, Madison, WI, USA
[7] Department of Data Science and AI, Wadhwani School of Data Science and AI, Indian Institute of Technology Madras, Chennai, TN, India
* Correspondence: karthik@bact.wisc.edu (Karthik Anantharaman)

This file includes:

- Supplementary Figures 1–26
- Supplementary Tables 1–6
- Descriptions of Supplementary Data 1–5
- Supplementary References

SUPPLEMENTARY FIGURES

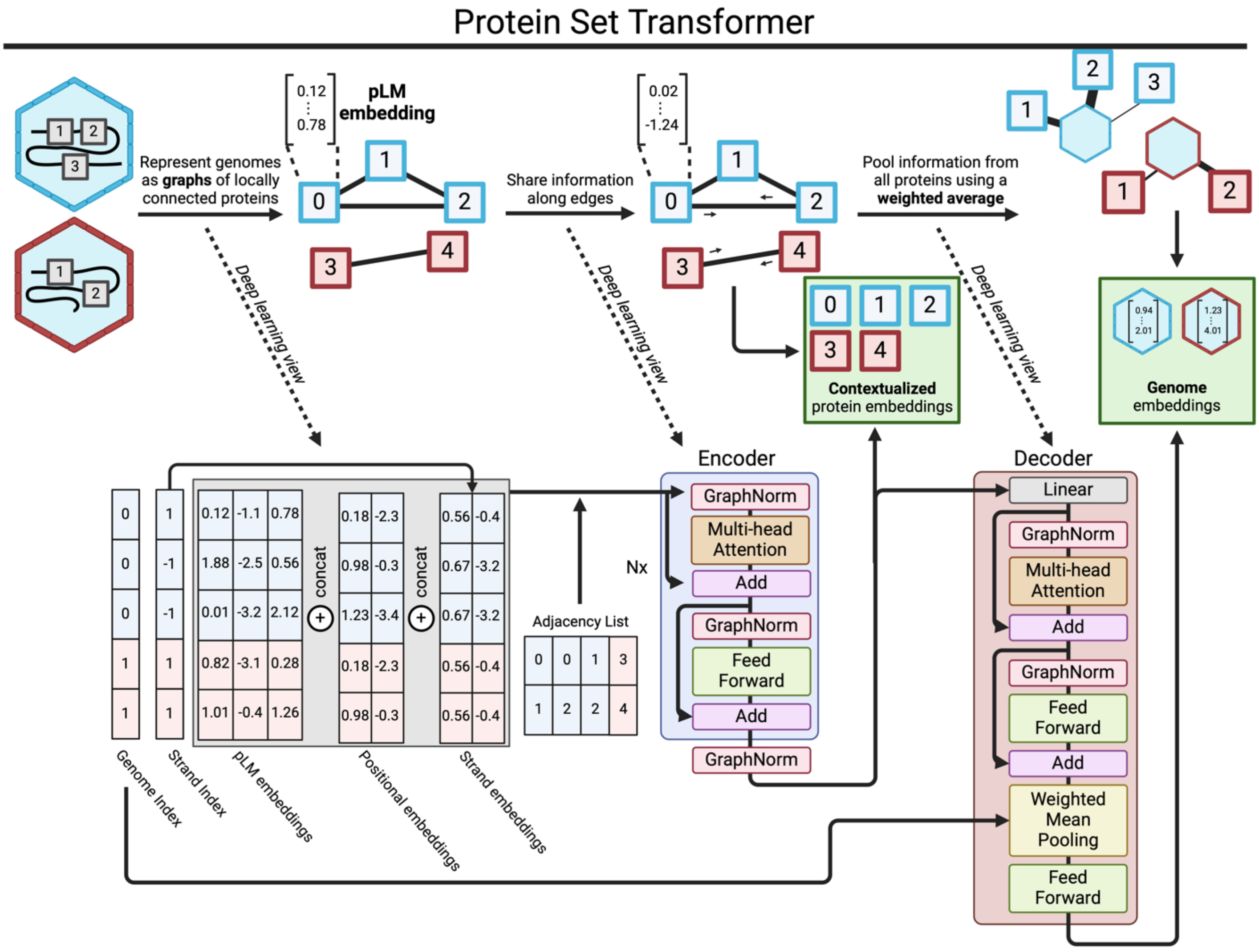

Supplementary Figure 1. A machine learning-centric view of the encoder-decoder Protein Set Transformer (PST) architecture. Each genome is internally represented as a graph, composed of subgraph genome chunks whose size is tunable. A minibatch of genomes is represented in a memory-efficient stacked matrix, where the boundaries for the set of proteins from each genome are tracked using indices and offset pointers for efficient random access. At the beginning of training, the ESM2 protein embeddings are concatenated with learnable positional embeddings based on the relative position in each genome and encoding strand embeddings. This is then input to the PST encoder, which uses multi-head attention for pairs of proteins defined by the initial adjacency matrix. This only allows each protein to attend to its neighbors in the same genome subgraph. The output from the PST encoder are genome-contextualized protein embeddings, which are also the inputs to the PST decoder. The PST decoder uses multi-head attention pooling to project each contextualized protein embedding onto a learnable seed vector. This learns weights for each protein, which are used to pool each protein representation into a final genome representation. The full encoder-decoder PST shown here is framework used by the PST-TL (triplet loss) models, which output both contextualized protein embeddings and genome embeddings that are learned weighted averages of the preceding protein embeddings. Encoder-only PSTs that only have protein-level objectives like PST-MLM (masked language modeling) models stop after the second step, only natively outputting contextualized protein embeddings. Genome embeddings for encoder-only PSTs can be generated with a simple average over proteins for each genome.

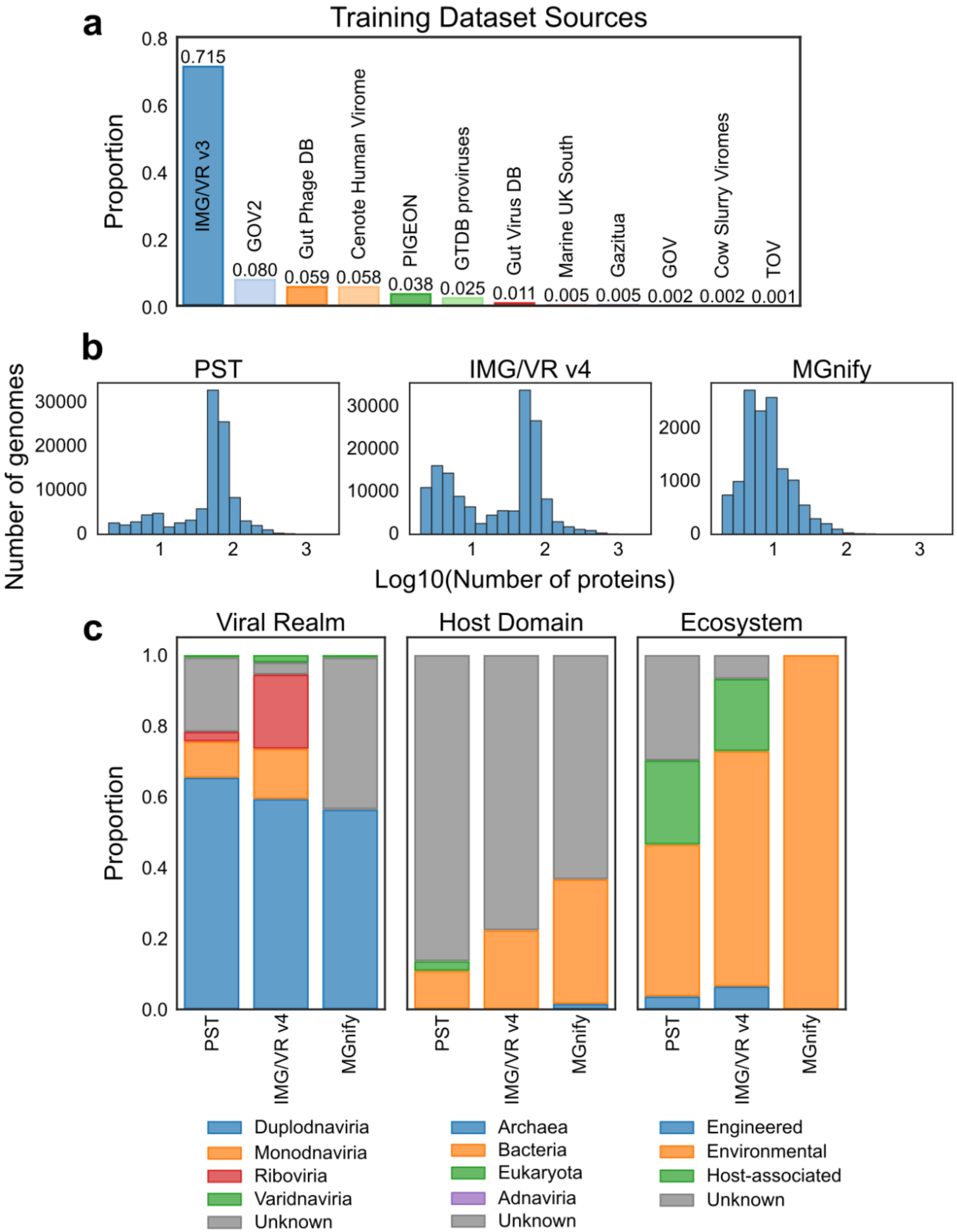

Supplementary Figure 2. Information about each dataset used for training and evaluation of PST. a Proportional source of 103,589 training set viral genomes (see Supplemental Table 1 for the source publication of each virus). **b** Distributions of the log10 number of proteins encoded per genome. **c** The relative distributions of viral realm, host domain, and broad ecosystem for each dataset. Viral realm, if not provided by the source database, was predicted by geNomad. For the PST training and IMG/VR v4 datasets, host domains that were not provided by the source database, excluding predicted proviruses, were considered unknown. For the MGnify test dataset, putative hosts were predicted by iPHoP.

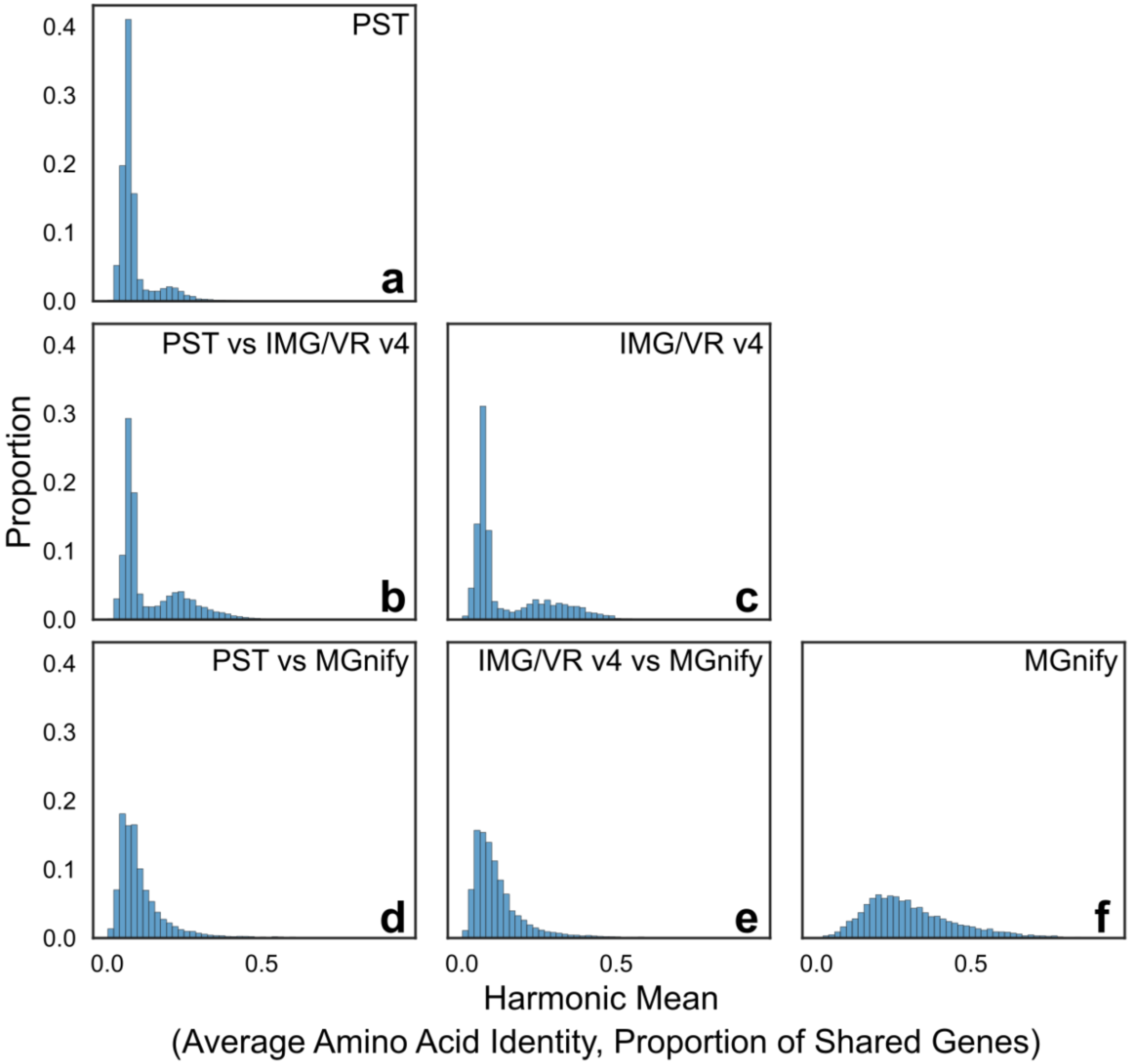

Supplementary Figure 3. Genome-genome similarity histograms for PST training and test datasets. Data shown compare the PST training dataset with itself (**a**), the PST training dataset with the IMG/VR v4 test dataset (**b**), the IMG/VR v4 test dataset with itself (**c**), the PST training dataset with the MGnify test dataset (**d**), the IMG/VR v4 test dataset with the MGnify test dataset (**e**), and the MGnify test dataset with itself (**f**). Genome-genome similarity was computed as the harmonic mean of the Average Amino Acid Identity and the proportion of shared genes between each pair of genomes.

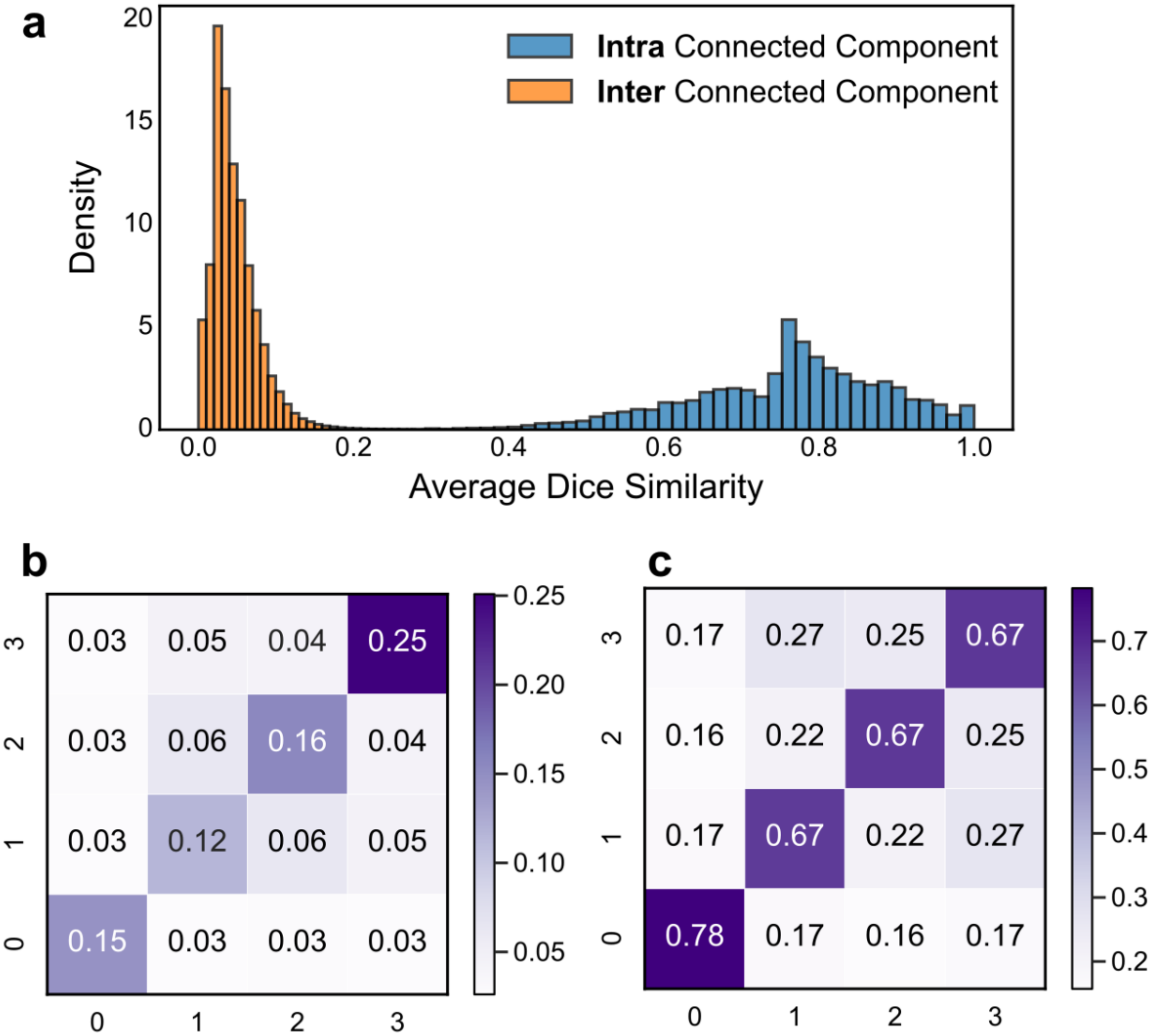

Supplementary Figure 4. Evaluation of 4 cross validation (CV) groups based on separating genomes into groups with minimally overlapping shared protein content. a The average dice score of all pairs of genomes in the same connected component (blue) and across connected components (orange) was plotted as a histogram. Dice scores were computed using protein cluster presence/absence binary matrices for all genomes (see **Methods**). **b** The average dice score between each of the 4 largest connected components used as seeds to expand each protein diversity CV group. **c** The average maximum dice score between each genome and all others from each of the 4 protein diversity CV groups. For **b, c**, the color bar is scaled based on the dice score values shown in the heatmaps.

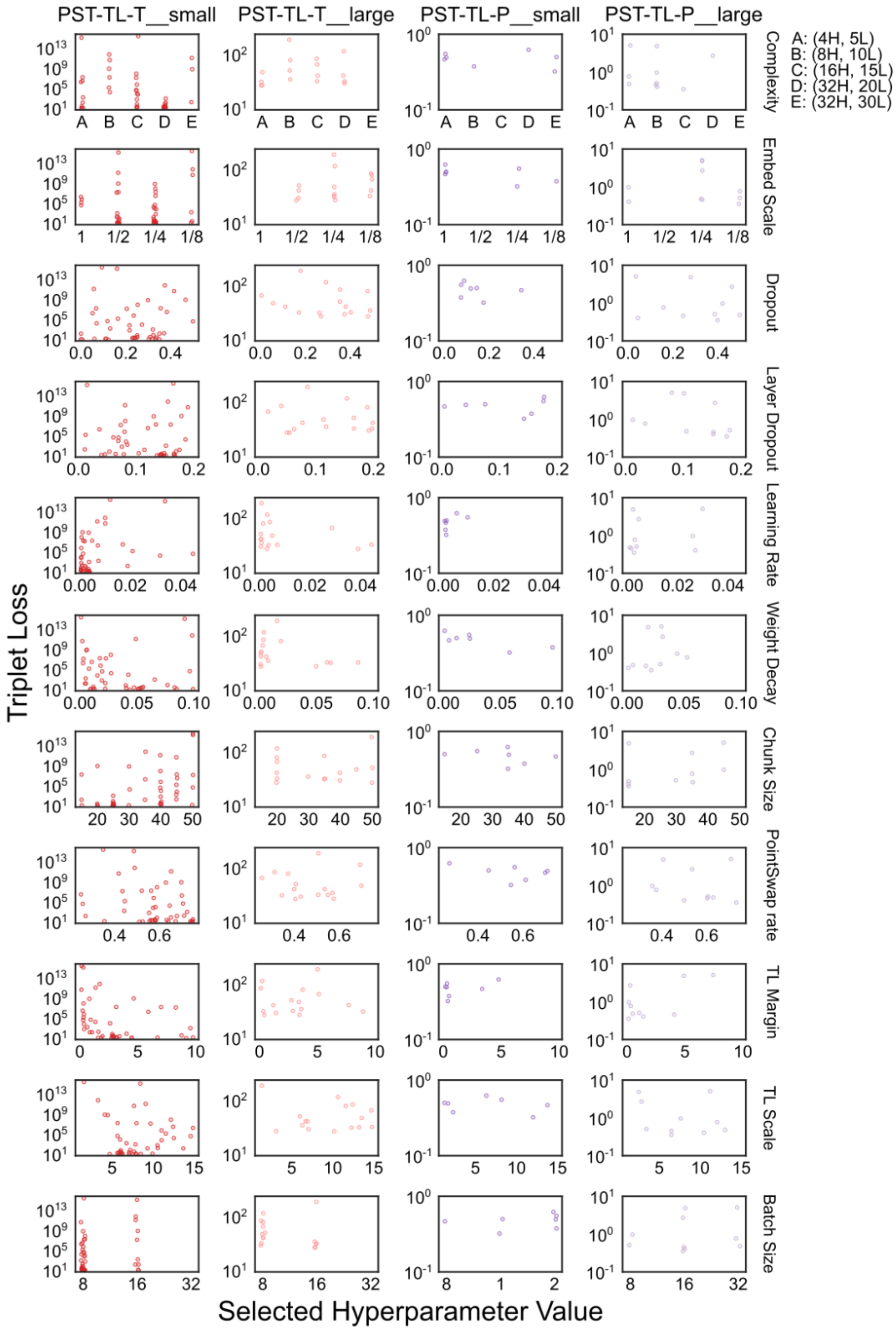

Supplementary Figure 5. Hyperparameter tuning for PST triplet loss models. Hyperparameters were tuned by cross validation based on taxonomic groups (T) or protein diversity groups (P). Rows indicate the specific hyperparameter, and columns indicate the model. There were 45 complete trials for PST-TL-T__small, 16 complete trials for PST-TL-T__large, 7 complete trials for PST-TL-P__small, and 10 complete trials for PST-TL-P__large. "Complexity" refers to the number of attention heads and encoder layers, with a key in the top right (H = attention heads, L = encoder layers, i.e. 4H = 4 attention heads, 5L = 5 encoder layers). "Embed scale" is the size of each of positional and strand embeddings relative to the input ESM2 protein embedding that are concatenated together. Weight decay is for the AdamW optimizer. The PST "chunk size" is the number of proteins per genome chunk. "PointSwap rate" is the proportion of proteins swapped between the anchor

and positive genome during PointSwap sampling. “TL margin” is the minimum margin between anchor-positive and anchor-negative embedding distances before the indicated triplet no longer contributes to the loss. “TL scale” is the negative exponential decay scale factor to adjust the weight of the choice of the negative samples in the triplet loss function. Batch size is in units of number of genomes.

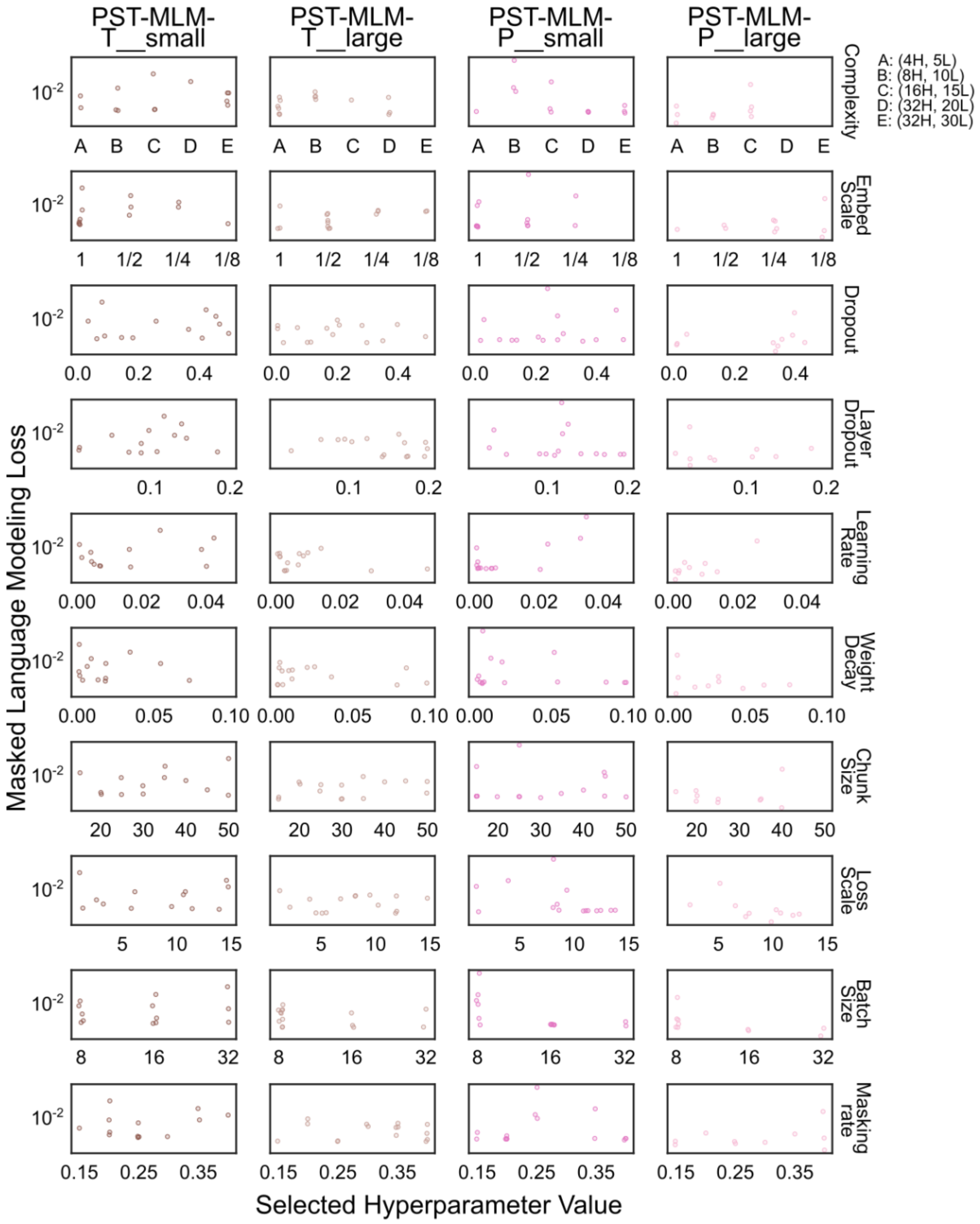

Supplementary Figure 6. Hyperparameter tuning for PST masked language modeling loss models. Hyperparameters were tuned by cross validation based on taxonomic groups (T) or protein diversity groups (P). Rows indicate the specific hyperparameter, and columns indicate the model. There were 13 complete trials for PST-MLM-T__small, 15 complete trials for PST-MLM-T__large, 15 complete trials for PST-MLM-P__small, and 10 complete trials for PST-MLM-P__large. "Complexity" refers to the number of attention heads and encoder layers, with a key in the top right (H = attention heads, L = encoder layers, i.e. 4H = 4 attention heads, 5L = 5 encoder layers). "Embed scale" is the size of each of positional and strand embeddings relative to the input ESM2 protein embedding that are concatenated together. Weight decay is for the AdamW optimizer. The PST "chunk size" is the number of proteins per genome chunk. "Loss scale" is the negative exponential decay scale factor to adjust the weight of the choice of the positive samples. Batch size is in units of number of genomes. "Masking rate" is the proportion of protein embeddings masked to 0-vectors per genome each time a minibatch is sampled.

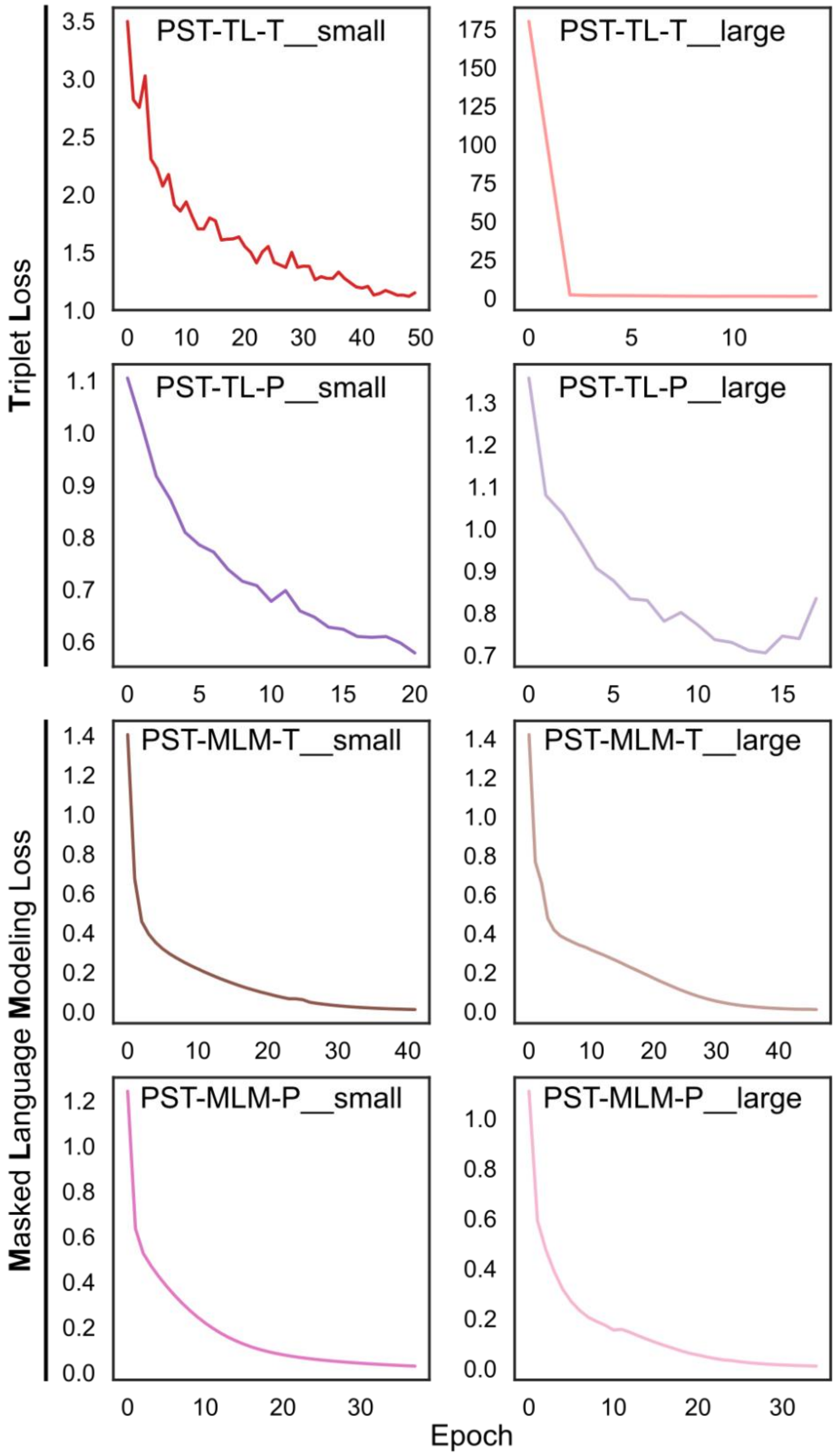

Supplementary Figure 7. Training loss curves for the final PST models after hyperparameters were chosen by cross validation. The names of each model indicate the loss objective (TL = Triplet Loss, MLM = Masked Language Modeling), how the cross validation groups were determined (T = viral taxonomic realm, P = protein diversity), and the size of the input ESM2 embeddings (small = esm2_t6_8M, large = esm2_t30_150M).

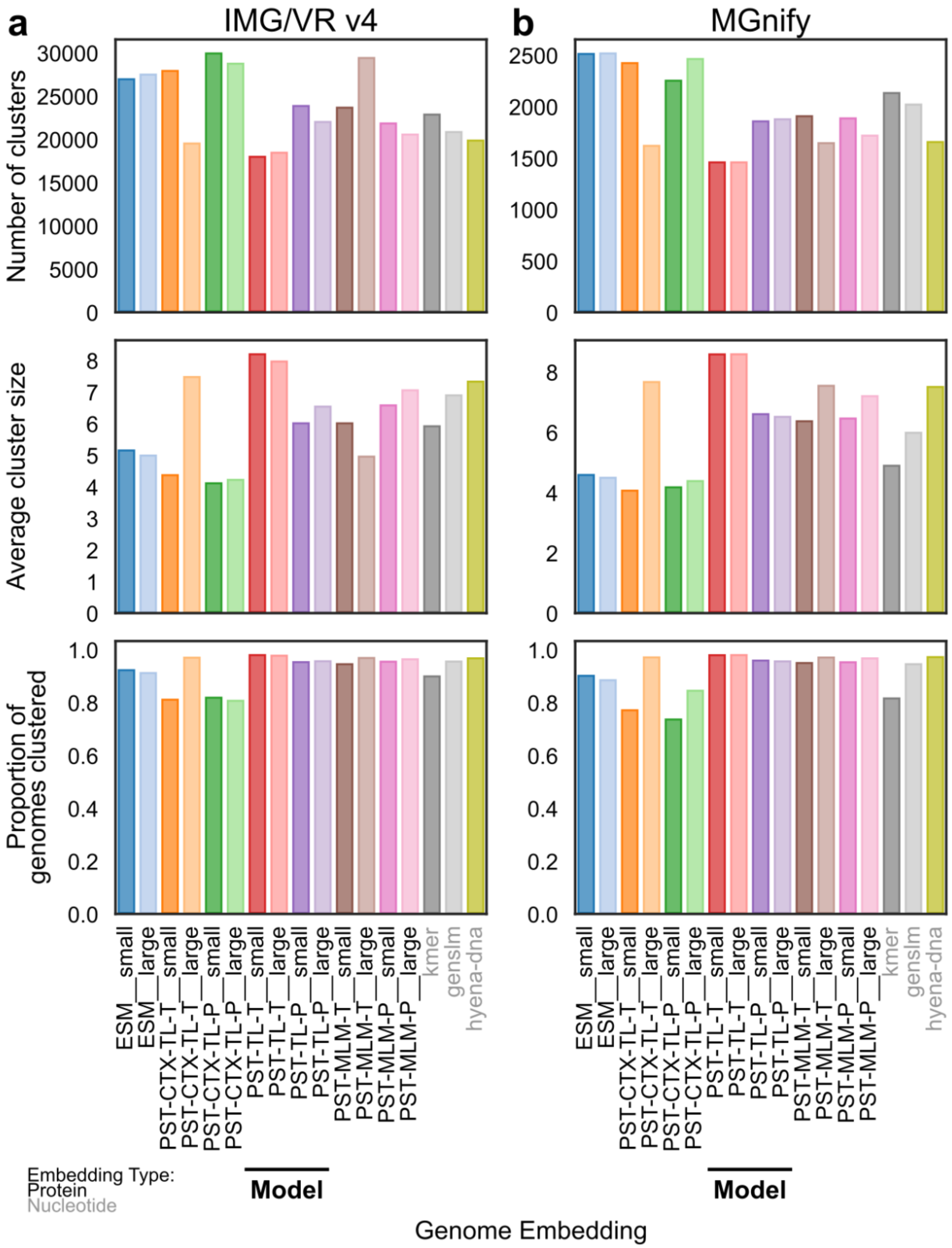

Supplementary Figure 8. Genome clustering stats. The test datasets are IMG/VR v4 (**a**) and MGnify (**b**). Genomes were clustered based on the angular similarity of L2-normalized genome embeddings from the corresponding embedding type on the x-axis. Singleton genomes not clustered were excluded from these stats.

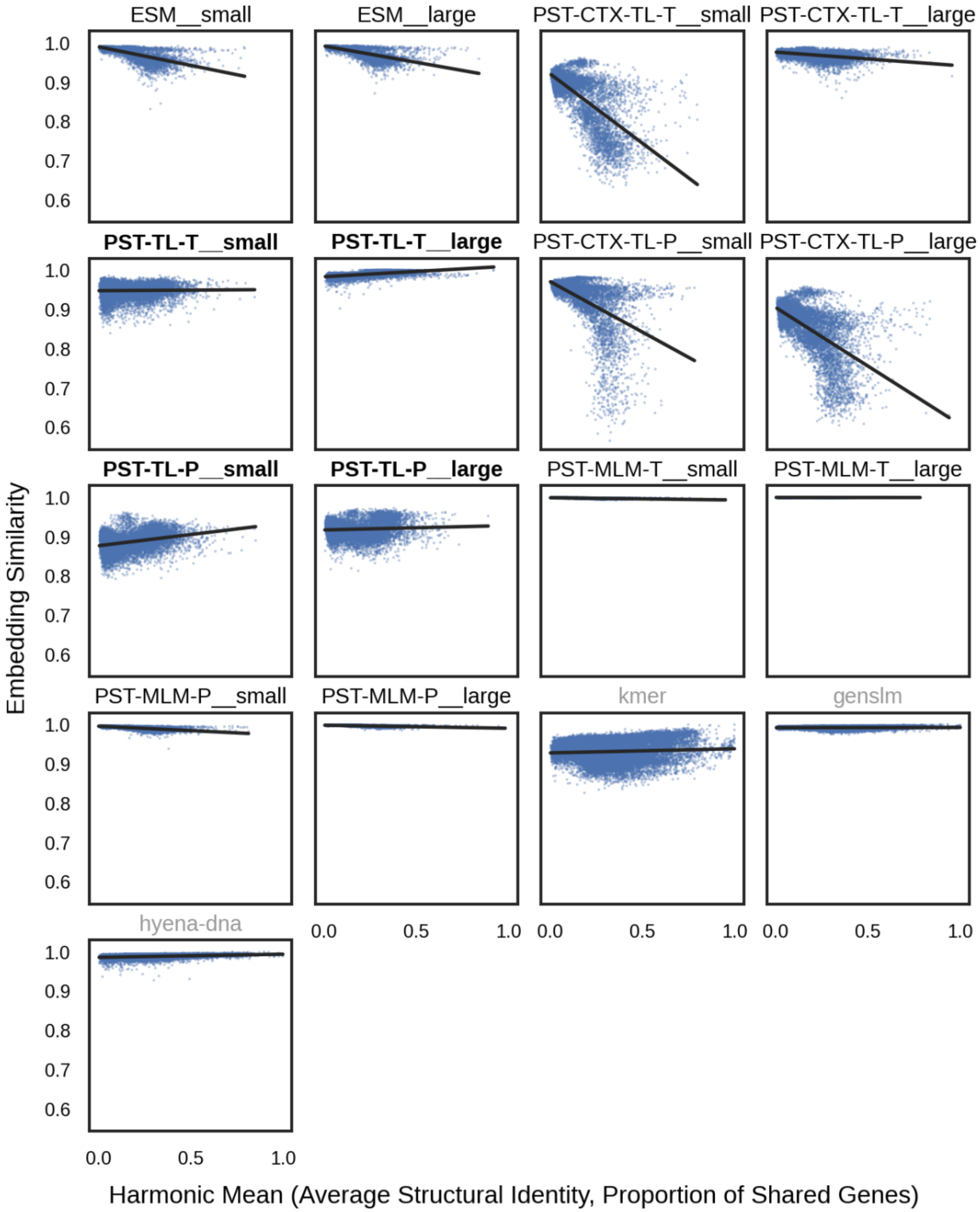

Supplementary Figure 9. Scatterplots of genome-genome similarity (x-axis) against embedding similarity (y-axis) for closely related genomes in the IMG/VR v4 test dataset. Genome-genome similarity is the harmonic mean of Average Structural Identity (ASI; see **Methods**) and the proportion of shared genes between each pair of genomes based on structural information. Each panel uses the corresponding genome embedding to compute the angular similarity between L2-normalized embeddings. ASI was only computed between genomes that clustered together based on the corresponding embedding. Genomes were defined as “similar” if there were any protein *sequence* alignments between the proteins from each pair of genomes.

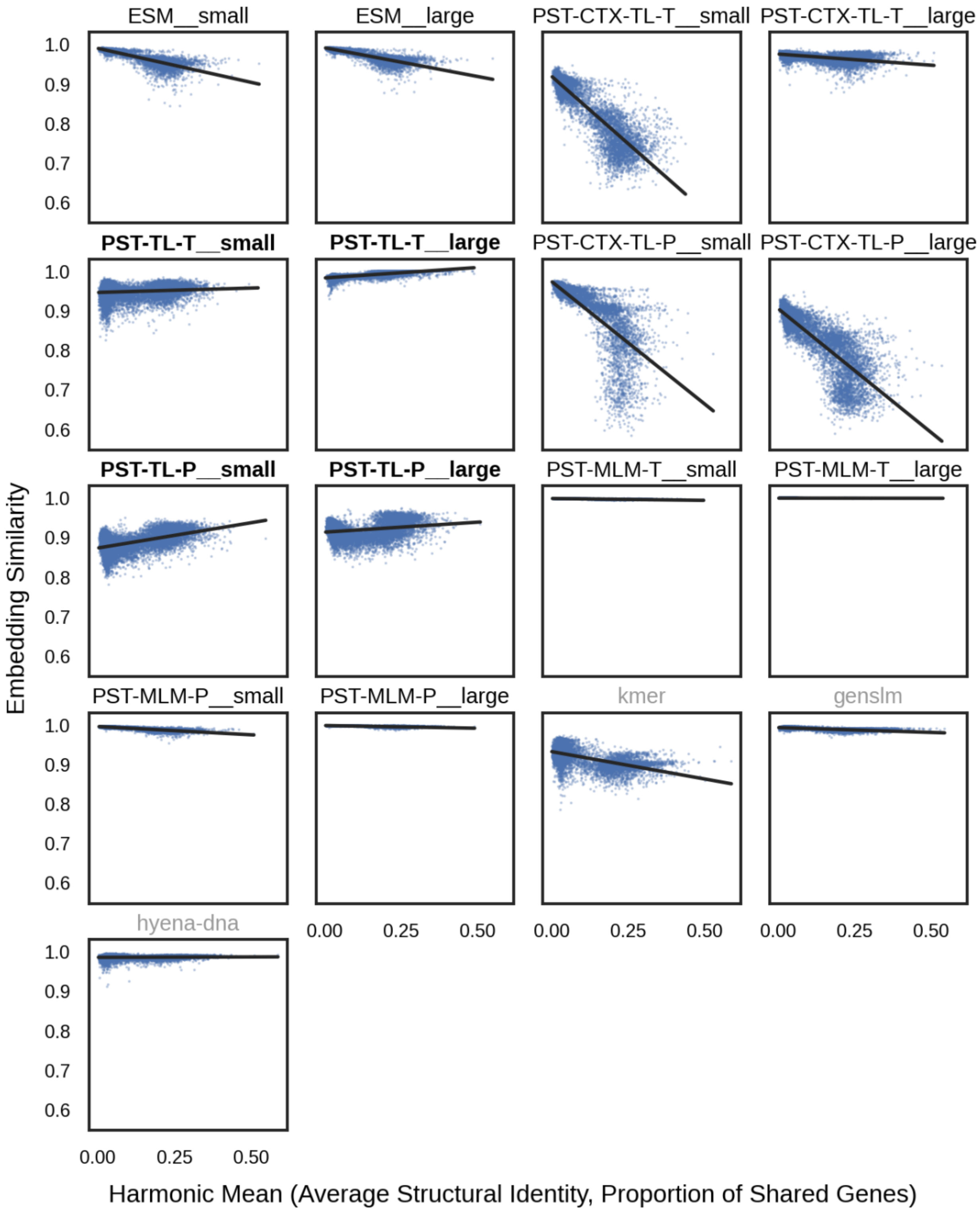

Supplementary Figure 10. Scatterplots of genome-genome similarity (x-axis) against embedding similarity (y-axis) for distantly related genomes in the IMG/VR v4 test dataset. Genome-genome similarity is the harmonic mean of Average Structural Identity (ASI; see **Methods**) and the proportion of shared genes between each pair of genomes based on structural information. Each panel uses the corresponding genome embedding to compute the angular similarity between L2-normalized embeddings. ASI was only computed between genomes that clustered together based on the corresponding embedding. Genomes were defined as “distant” if there were no protein *sequence* alignments and only protein *structural* alignments between the proteins from each pair of genomes.

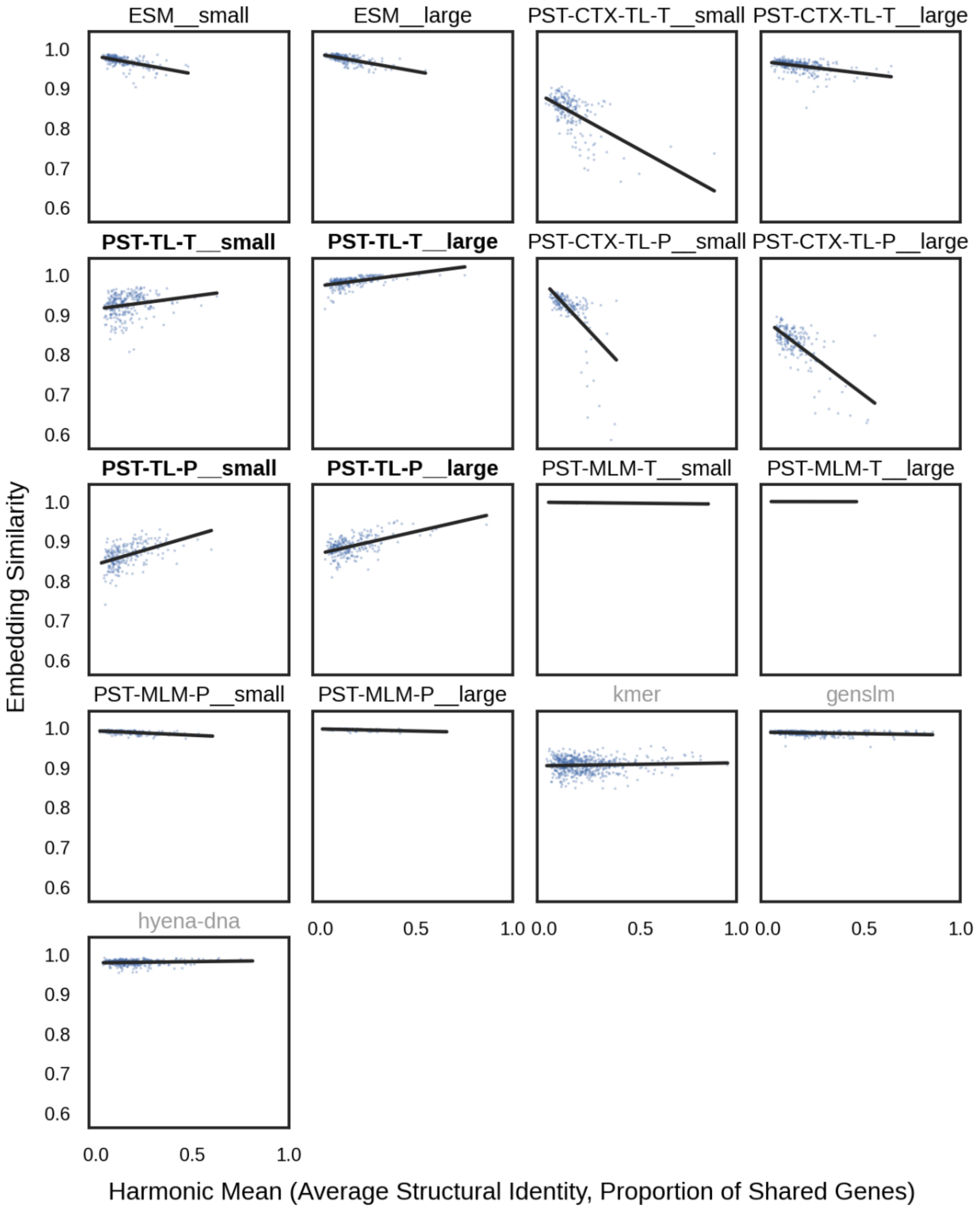

Supplementary Figure 11. Scatterplots of genome-genome similarity (x-axis) against embedding similarity (y-axis) for closely related genomes in the MGnify test dataset. Genome-genome similarity is the harmonic mean of Average Structural Identity (ASI; see **Methods**) and the proportion of shared genes between each pair of genomes based on structural information. Each panel uses the corresponding genome embedding to compute the angular similarity between L2-normalized embeddings. ASI was only computed between genomes that clustered together based on the corresponding embedding. Genomes were defined as "similar" if there were any protein *sequence* alignments between the proteins from each pair of genomes.

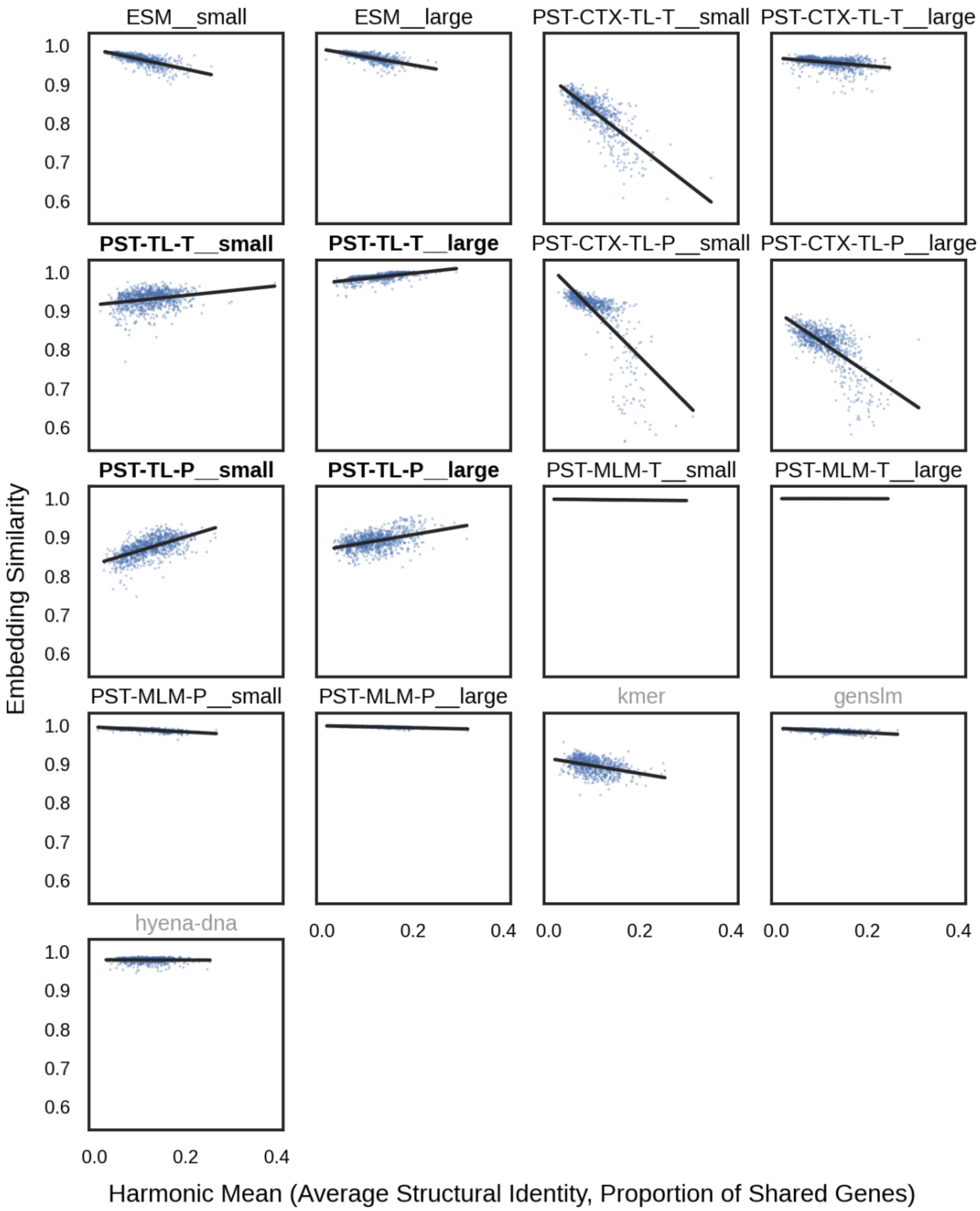

Supplementary Figure 12. Scatterplots of genome-genome similarity (x-axis) against embedding similarity (y-axis) for distantly related genomes in the MGnify test dataset. Genome-genome similarity is the harmonic mean of Average Structural Identity (ASI; see **Methods**) and the proportion of shared genes between each pair of genomes based on structural information. Each panel uses the corresponding genome embedding to compute the angular similarity between L2-normalized embeddings. ASI was only computed between genomes that clustered together based on the corresponding embedding. Genomes were defined as "distant" if there were no protein *sequence* alignments and only protein *structural* alignments between the proteins from each pair of genomes.

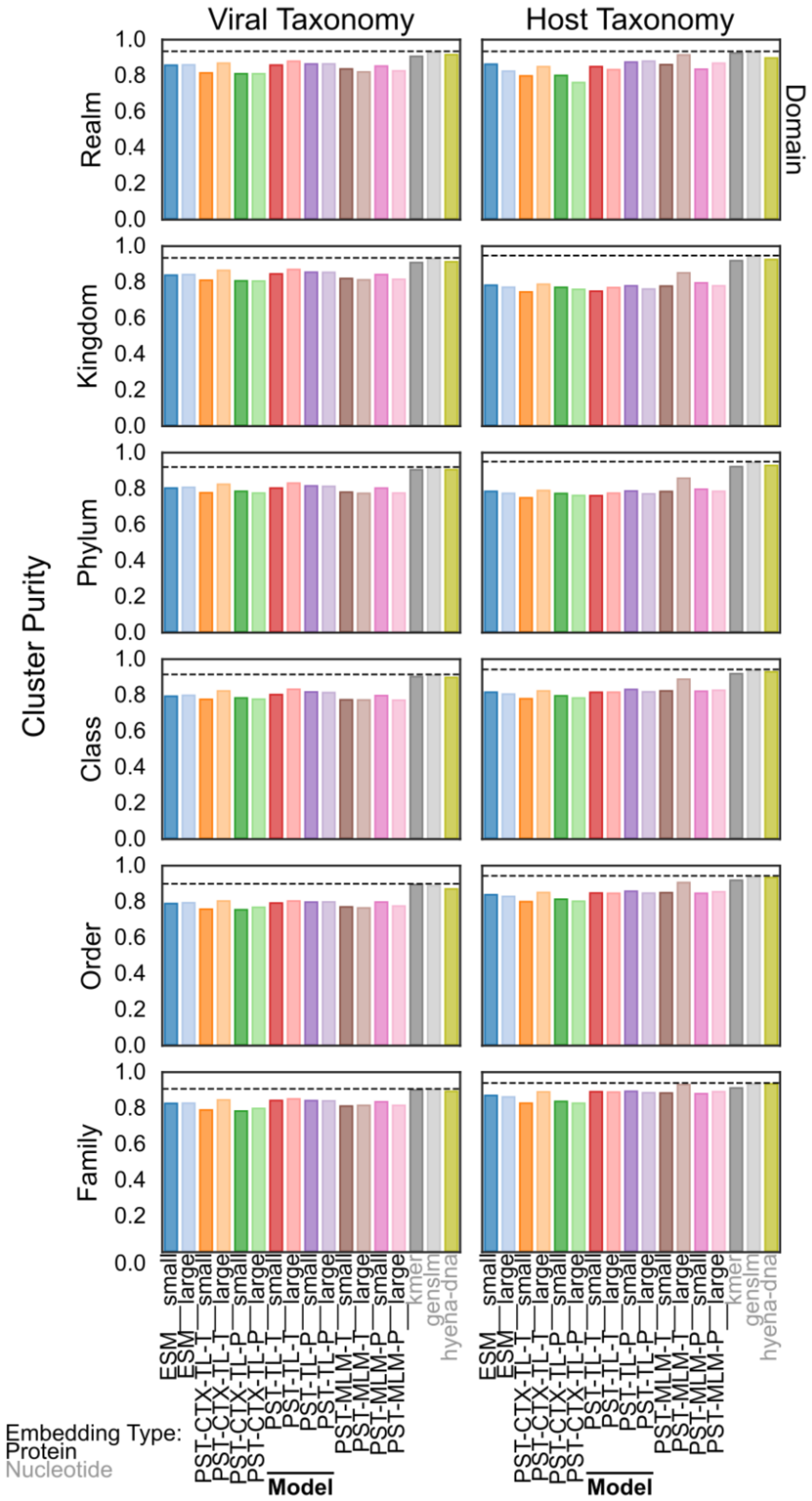

Supplementary Figure 13. Viral and Host taxonomic purity for the IMG/VR v4 test dataset. Purity is defined as the cluster size-weighted average of information gain ratio for non-singleton genome clusters. Missing or unknown taxonomic labels were excluded in clusters that had at least 1 labeled genome.

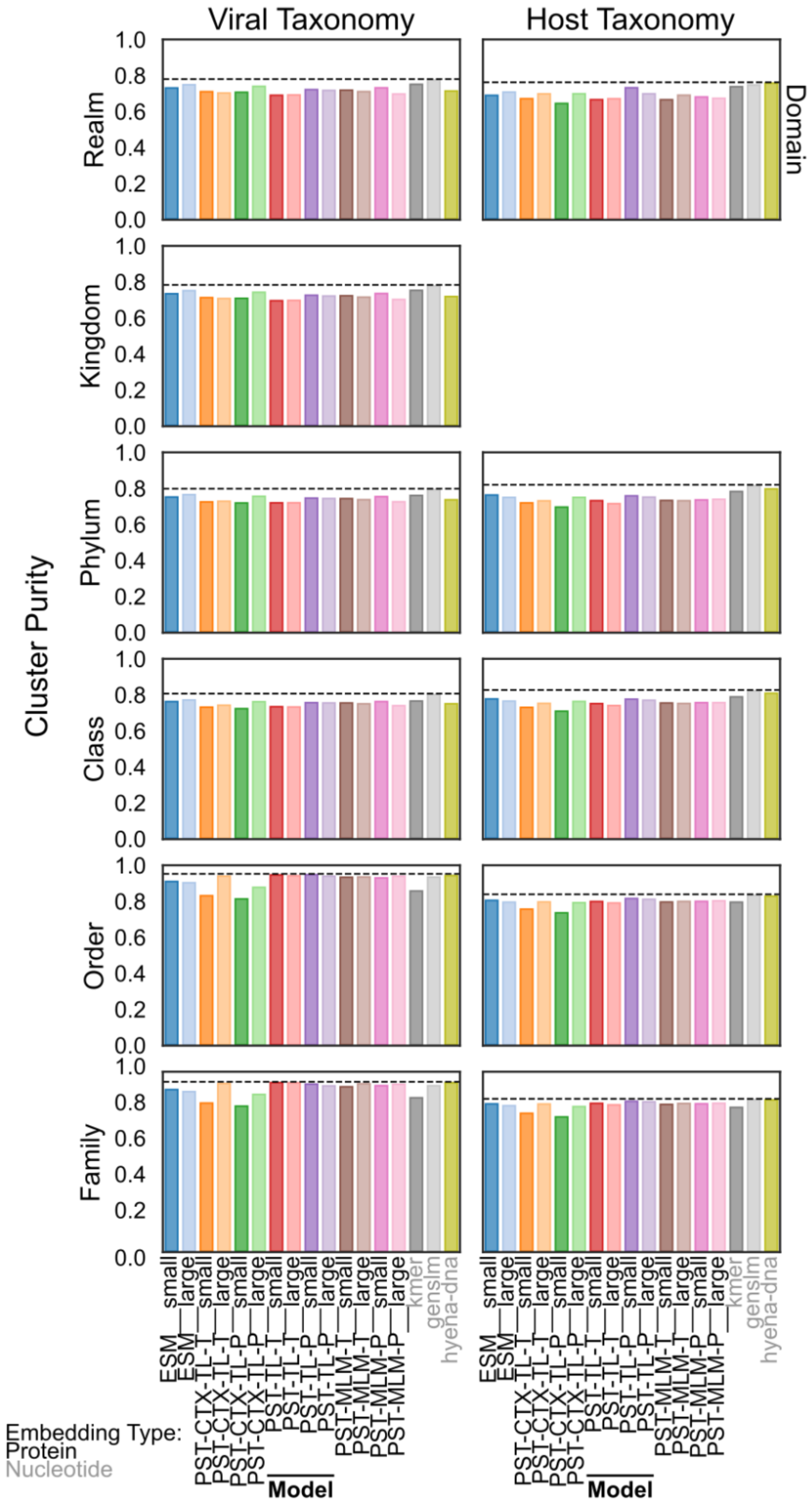

Supplementary Figure 14. Viral and Host taxonomic purity for the MGnify test dataset. Purity is defined as the cluster size-weighted average of information gain ratio for non-singleton genome clusters. Missing or unknown taxonomic labels were excluded in clusters that had at least 1 labeled genome. Host taxonomic labels were predicted by iPHoP, which does not output a predicted host kingdom even when it could be inferred from the domain.

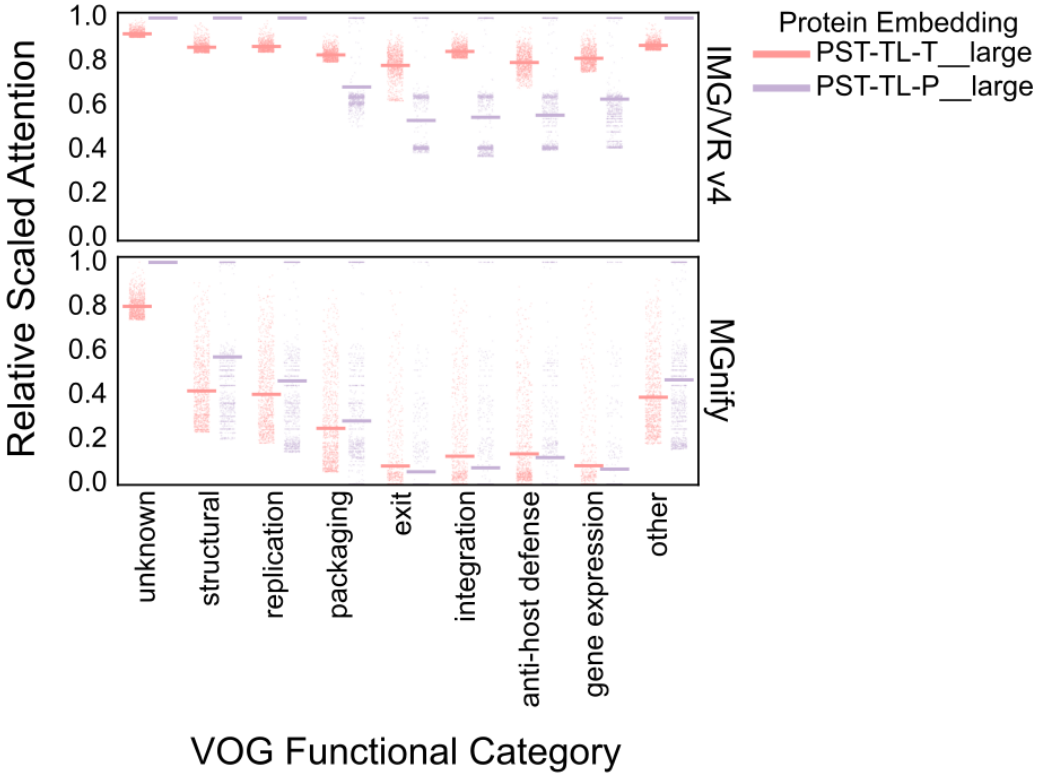

Supplementary Figure 15. PST decoder attention loosely correlates with protein function. Relative scaled attention from large triplet loss PSTs for proteins belonging to different VOG functional categories for IMG/VR v4 (top) and MGnify (bottom) test datasets. The per-protein attention scores from each model were normalized by the number of proteins encoded per scaffold (see **Methods**). For each model, these normalized attention values were then rescaled so that the maximum value was 1. Then the top 1,000 proteins based on the rescaled normalized attention were chosen for each functional category. Dots are the relative scaled attention value for one of the n=1,000 proteins chosen from each category, and the horizontal bars are the mean.

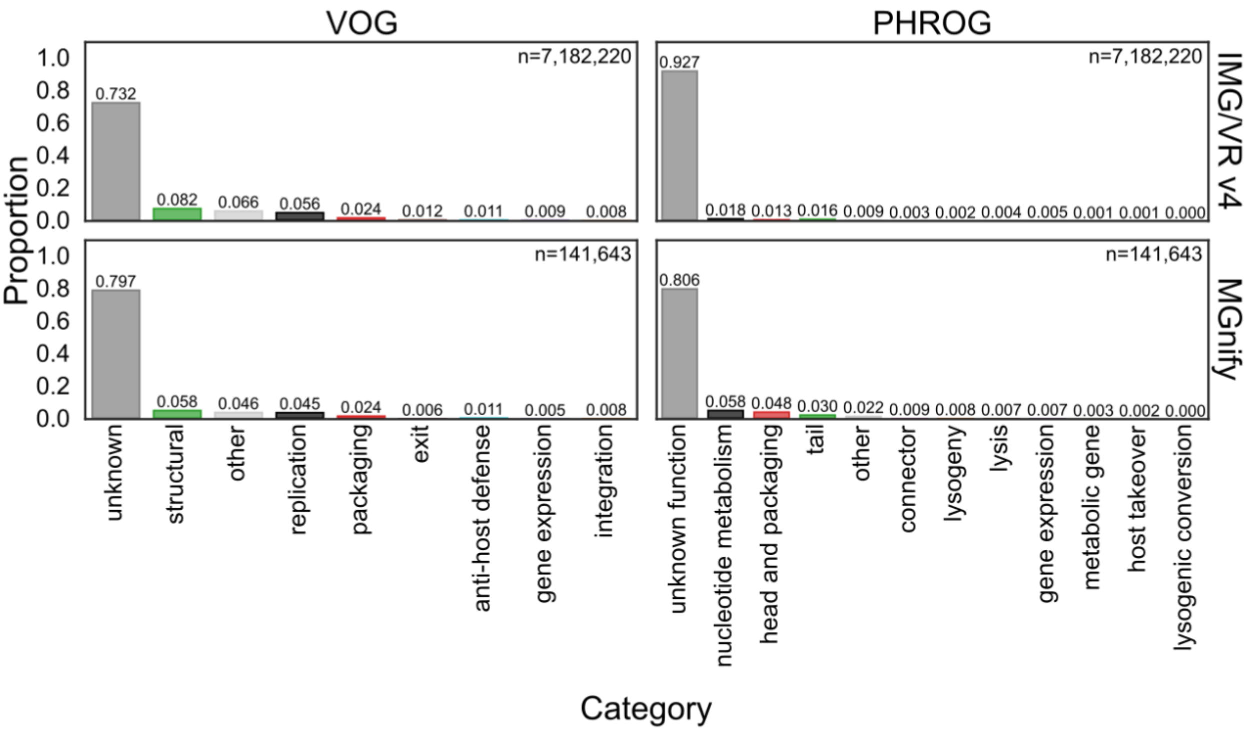

Supplementary Figure 16. Distribution of protein functional annotations. Annotations used curated categories from the VOG (left) or PHROG (right) databases for the proteins from the IMG/VR v4 (top) and MGnify (bottom) test datasets.

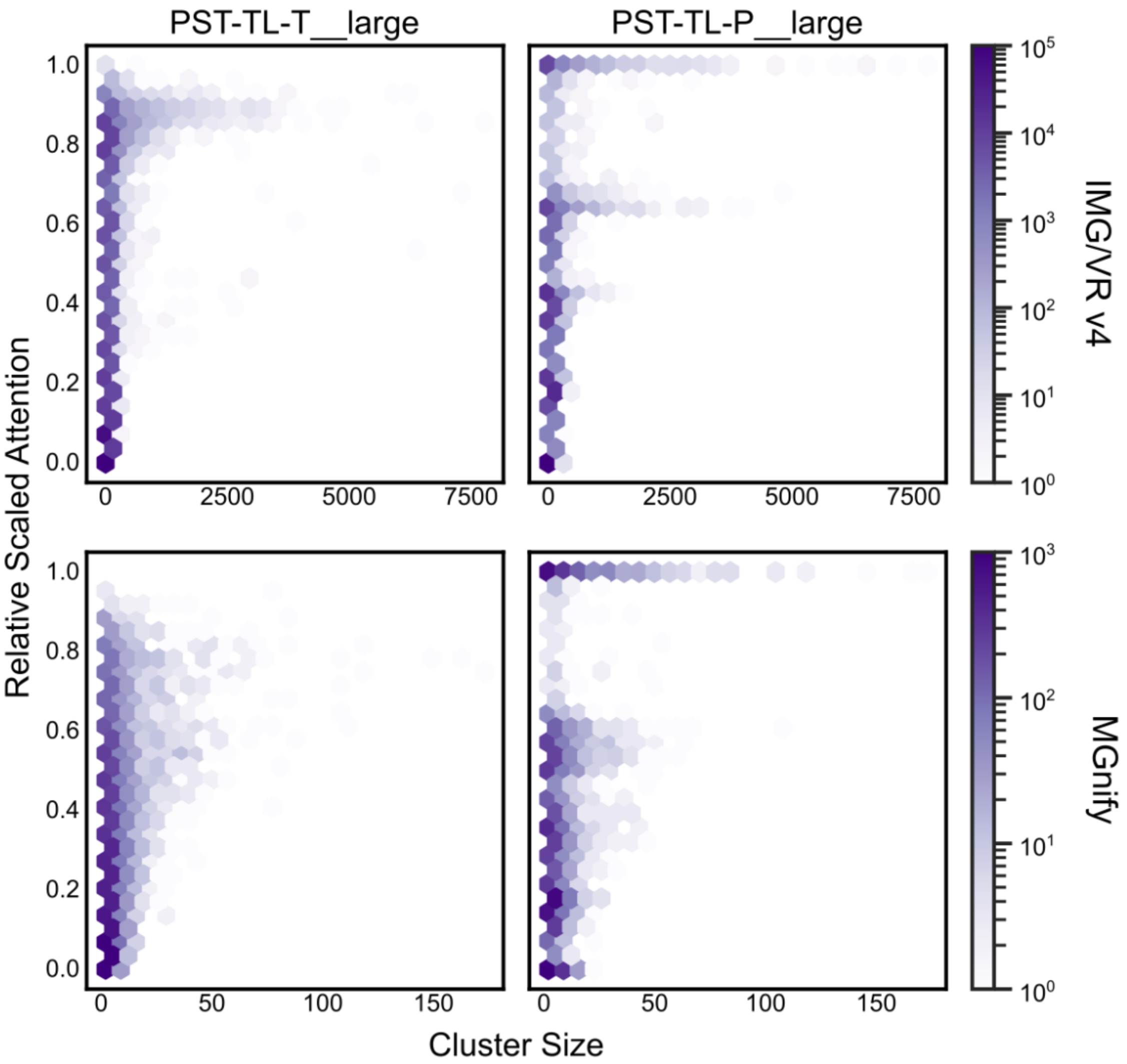

Supplementary Figure 17. PST decoder attention loosely correlates with sequence identity cluster size. Relative scaled attention from large triplet loss PSTs compared against the size of sequence identity-based clusters. The per-protein attention scores from each model were normalized by the number of proteins encoded per scaffold (see **Methods**). For each model, these normalized attention values were then rescaled so that the maximum value was 1. Then, for each protein cluster, the maximum scaled normalized attention was selected for the hexagonal histograms. The shade indicates the number of proteins in each bin (log scale).

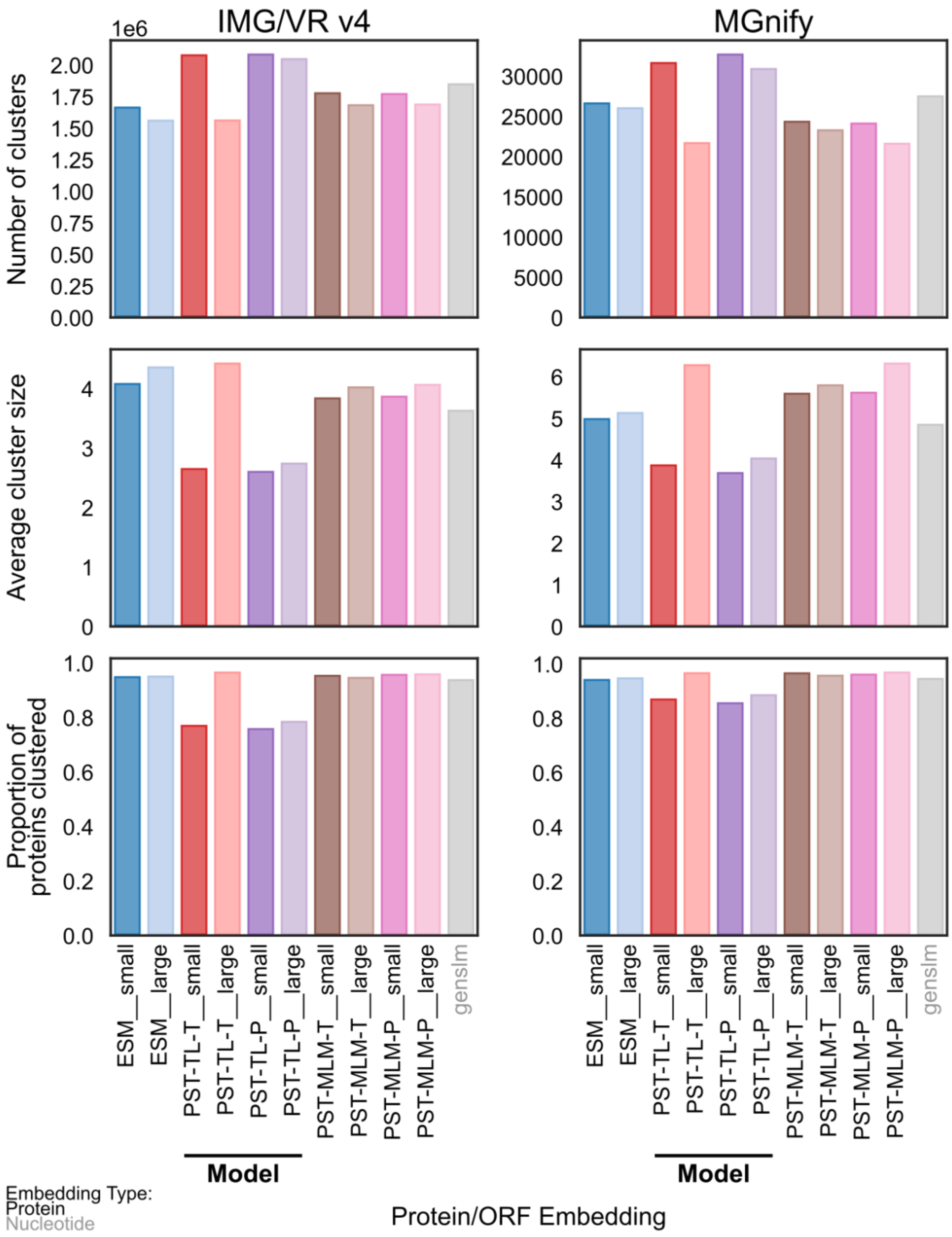

Supplementary Figure 18. Protein clustering stats for the IMG/VR v4 and MGnify test datasets. Proteins were clustered based on the angular similarity of L2-normalized protein embeddings from the corresponding embedding type on the x-axis. Singleton proteins not clustered were excluded from these stats.

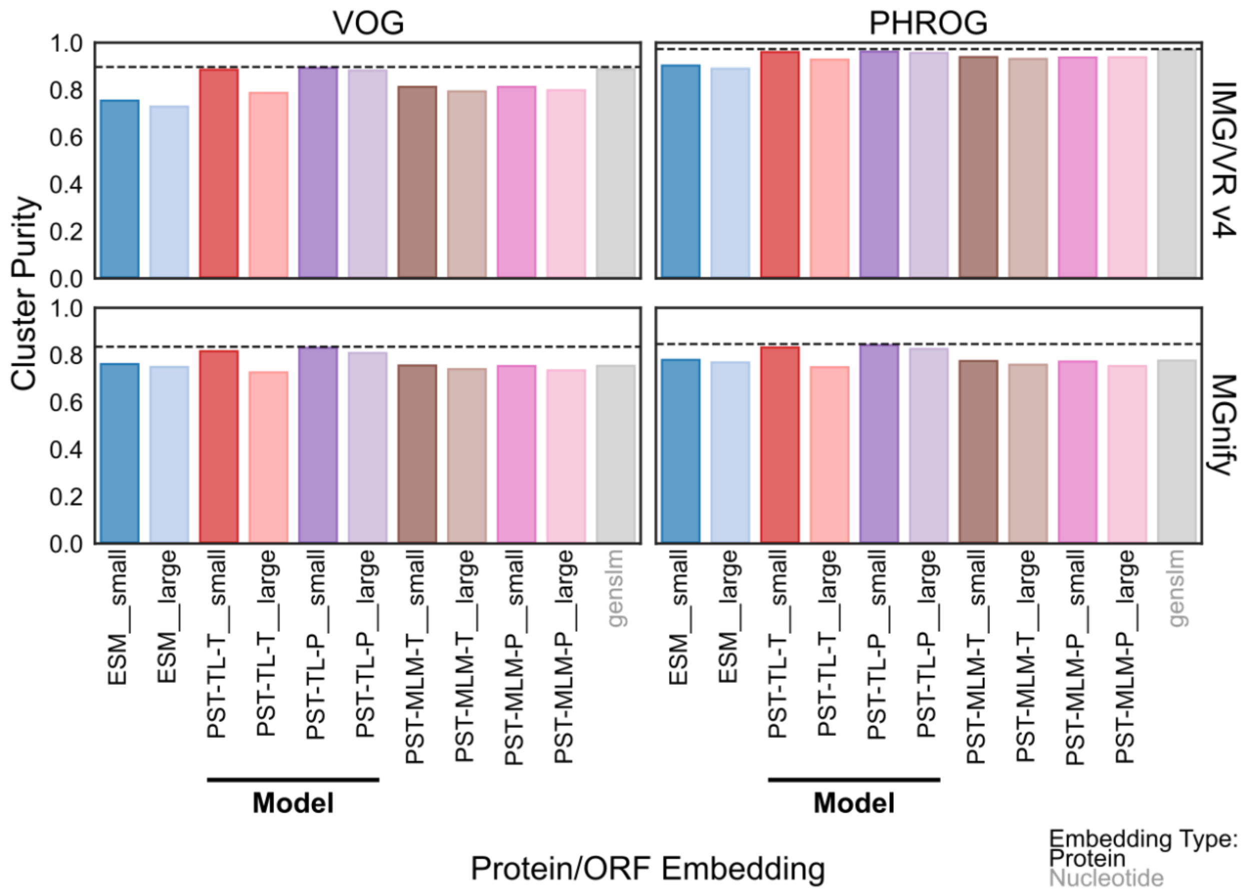

Supplementary Figure 19. Protein cluster functional purity. The IMG/VR v4 (top) and MGnify (bottom) test datasets were annotated with the VOG (left) or PHROG (right) databases. Proteins were clustered based on the angular similarity of L2-normalized protein embeddings from the corresponding embedding type on the x-axis. Only clusters that had at least 1 labeled protein were included, and in these clusters, all proteins without a predicted function were ignored. Purity is defined as the cluster size-weighted average of information gain ratio for these protein clusters.

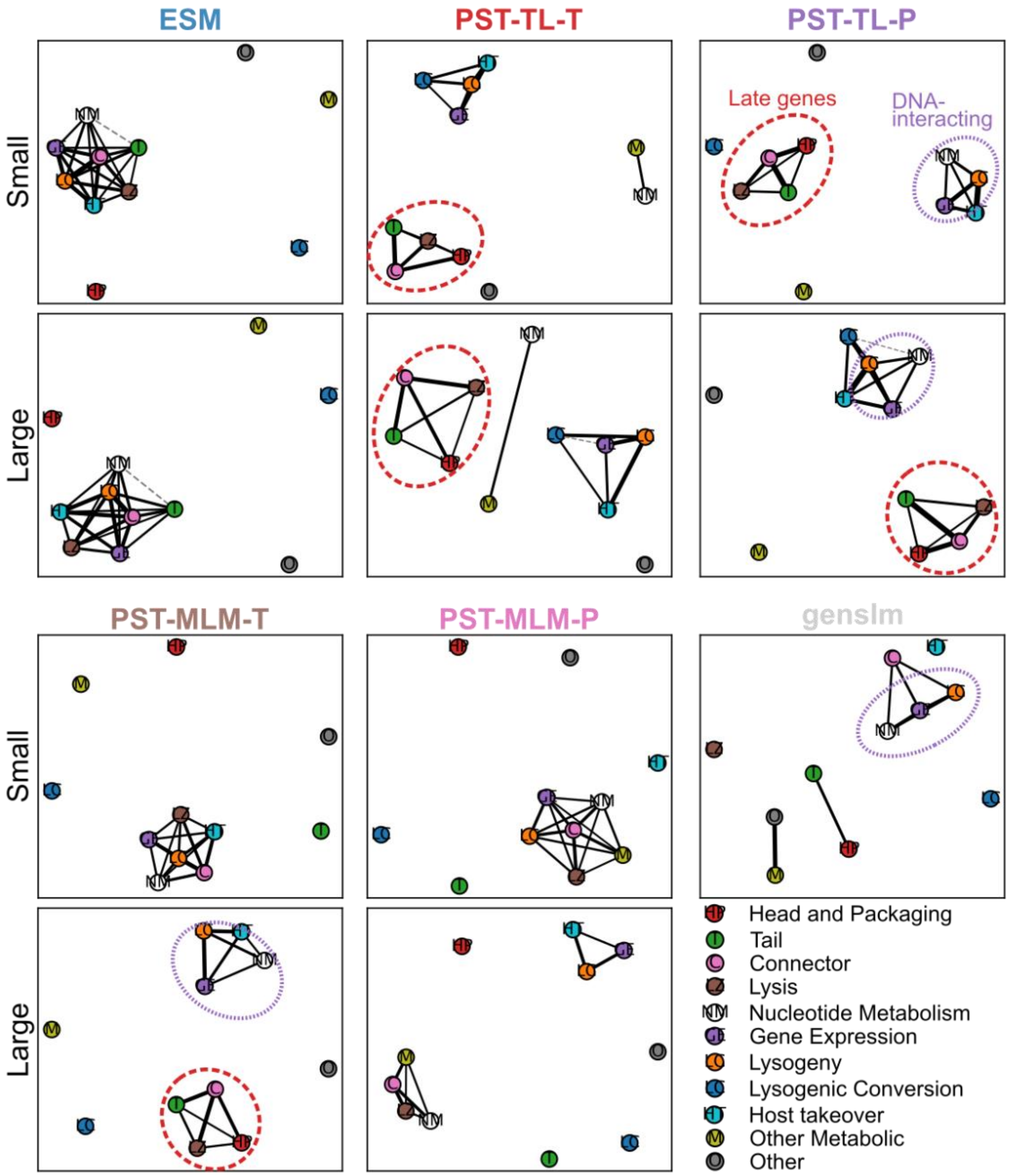

Supplementary Figure 20. Protein function co-clustering summary for the IMG/VR v4 test dataset based on PHROG annotations. Rows indicate the model size (i.e. the first panel is "ESM__small", excluding GenSLM), and columns indicate the protein embedding type. Each connected component was determined by Leiden community detection in co-clustering graph with a resolution of 1.25 (see **Methods**). Edges indicate functional categories that were more enriched in protein clusters compared to the joint occurrence of these categories in the PHROG database. The length of the edges inversely indicate the degree of enrichment (i.e. shorter edge = more enriched). Dashed gray edges indicate connections that were less enriched than expected but still clustered together. Red dashed circles highlight detected late gene modules, composed of Head and Packaging, Tail, Connector, and Lysis. Purple dotted circles indicate detected DNA-interacting modules, consisting of Nucleotide Metabolism, Gene Expression, and Lysogeny.

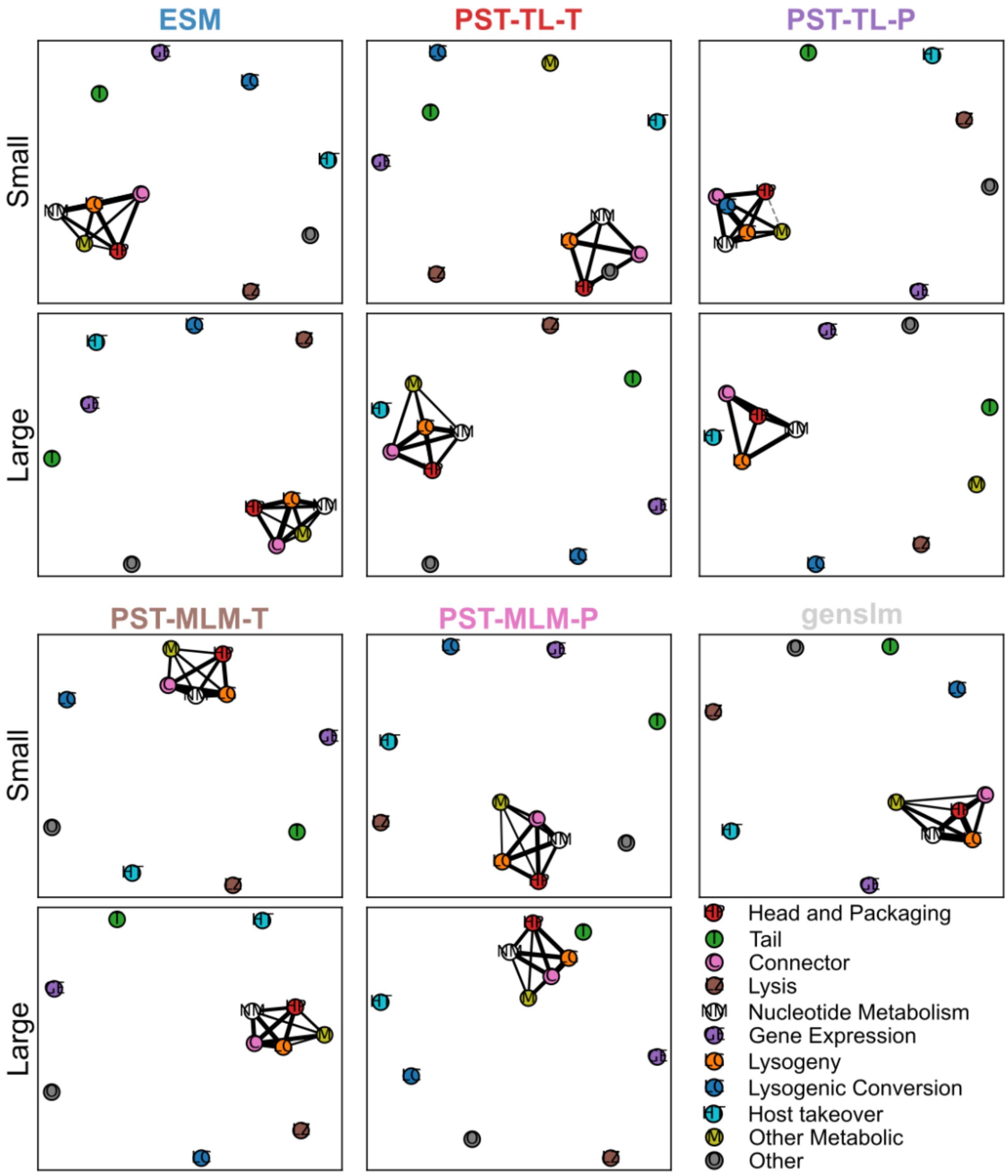

Supplementary Figure 21. Protein function co-clustering summary for the MGnify test dataset based on PHROG annotations. Rows indicate the model size (i.e. the first panel is "ESM__small", excluding GenSLM), and columns indicate the protein embedding type. Each connected component was determined by Leiden community detection in co-clustering graph with a resolution of 1.25 (see **Methods**). Edges indicate functional categories that were more enriched in protein clusters compared to the joint occurrence of these categories in the PHROG database. The length of the edges inversely indicate the degree of enrichment (i.e. shorter edge = more enriched). Dashed gray edges indicate connections that were less enriched than expected but still clustered together.

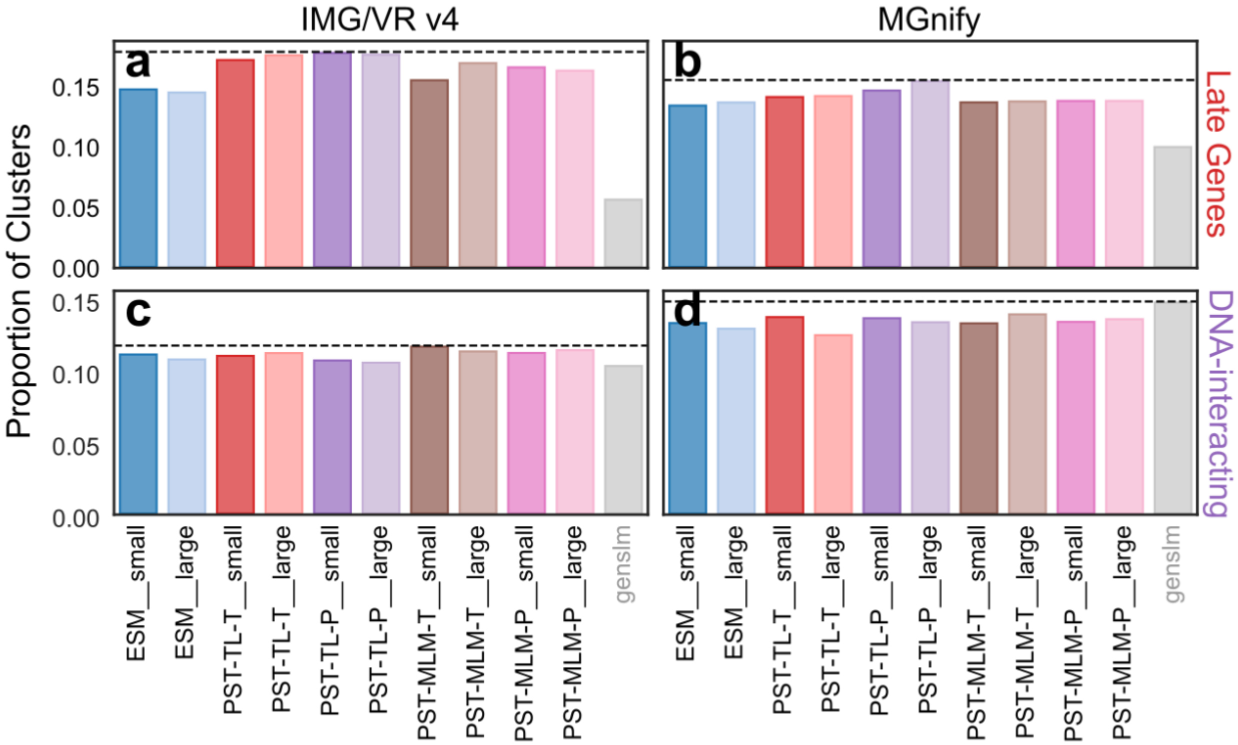

Supplementary Figure 22. Functional module detection. The proportion of protein clusters that correspond to Late Gene (**a, b**) or DNA-interacting (**c, d**) modules for the IMG/VR v4 (**a, c**) and MGnify (**b, d**) test datasets. A protein cluster was considered to represent a functional module if all proteins annotated by VOG in the cluster belonged to the subcategories that composed each module (Late Genes: Structural, Exit, Packaging; DNA-interacting: Replication, Integration, Packaging, Gene Expression). Each protein cluster was also required to have annotated proteins belonging to at least 2 of the subcategories.

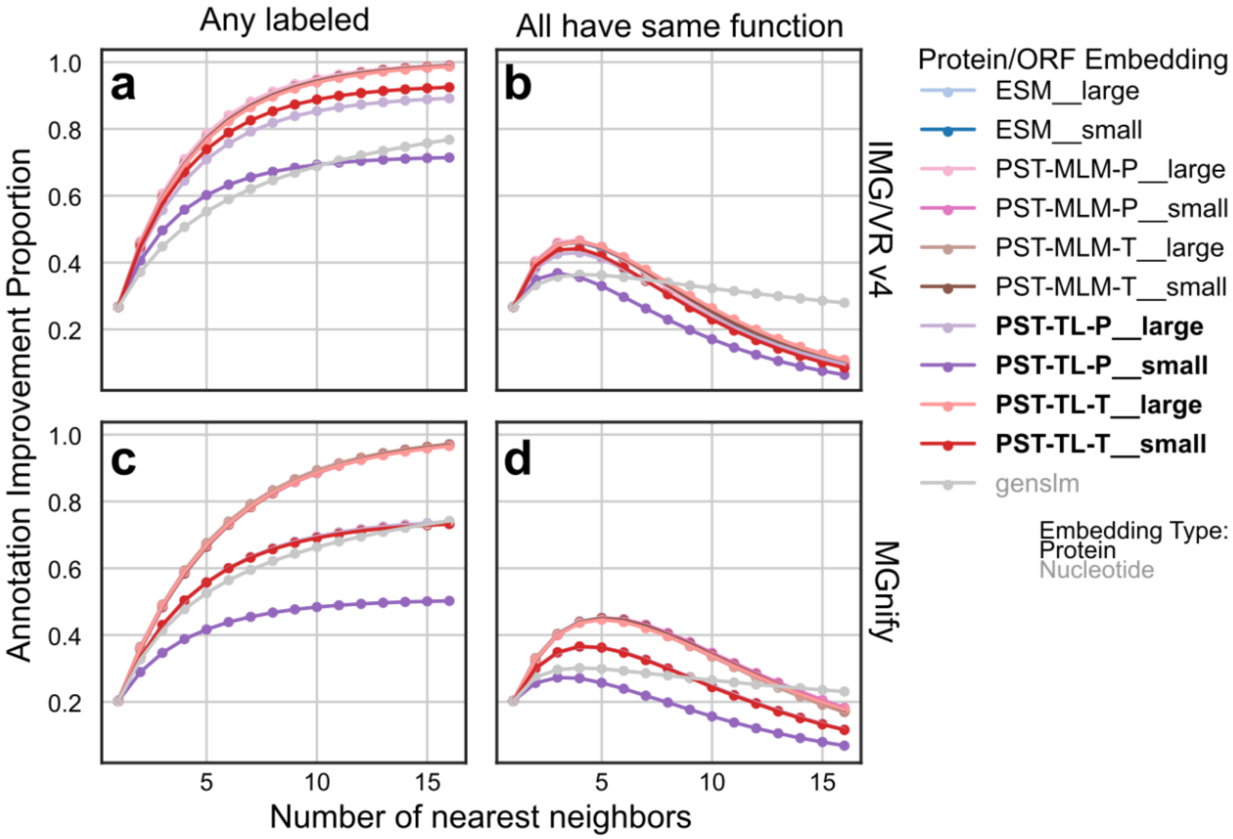

Supplementary Figure 23. Embedding-based annotation transfer. The proportion of proteins unannotated by VOG whose nearest neighbors in embedding space are annotated. The colors indicate the protein/ORF embedding. Nearest neighbors were searched after L2-normalizing the protein embeddings using angular similarity. **a, c** A hit was considered for each unannotated protein if any of the neighbors less than or equal to the current number of nearest neighbors were annotated. **b, d** A hit was considered similarly to (**a, c**) with the additional constraint that all of the current set of nearest neighbors must belong to the same VOG functional category. Unannotated proteins were not used to penalize the score. The IMG/VR v4 test dataset was used for (**a, b**), and the MGnify test dataset was used for (**c, d**).

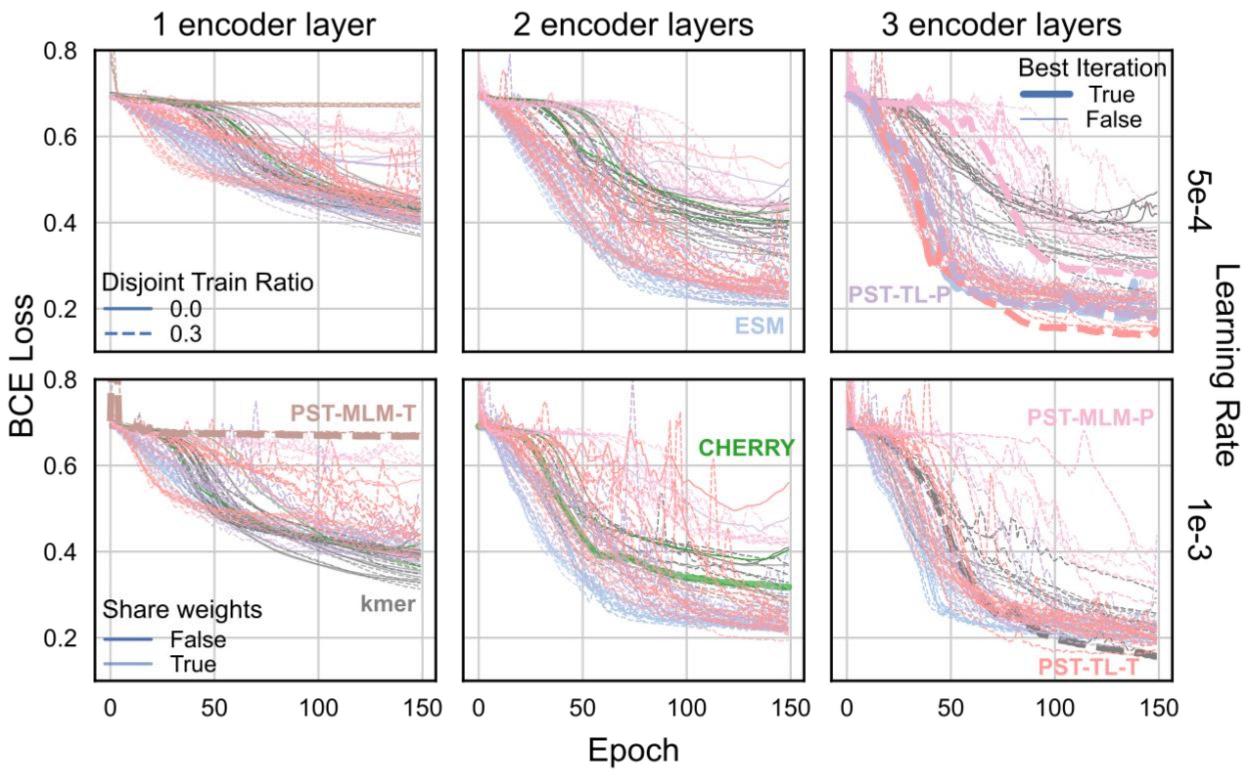

Supplementary Figure 24. Host prediction model training curves. Binary cross entropy loss curves for graph-based host prediction models. Columns indicate the number of encoder layers, and rows indicate the learning rate for the AdamW optimizer. Colors indicate the genome embedding used for the node embeddings of both virus and host genomes. For all embedding types except CHERRY and kmer, the indicated genome embedding was also used to cluster viruses to create virus-virus edges. Bold lines indicate the model iteration that was chosen based on the minimum loss during the final 20 epochs. Solid / dashed lines indicate the disjoint training ratio, which allocates a certain proportion of edges for a validation set during training. In the case of 0.0, all training edges were used for message passing and inference. The opacity of the lines indicate if the model weights were shared between the virus-host and virus-virus edges during message passing in the same layer. Only curves with a Spearman correlation ≤-0.9 were included to select iterations where the loss was strongly monotonically decreasing.

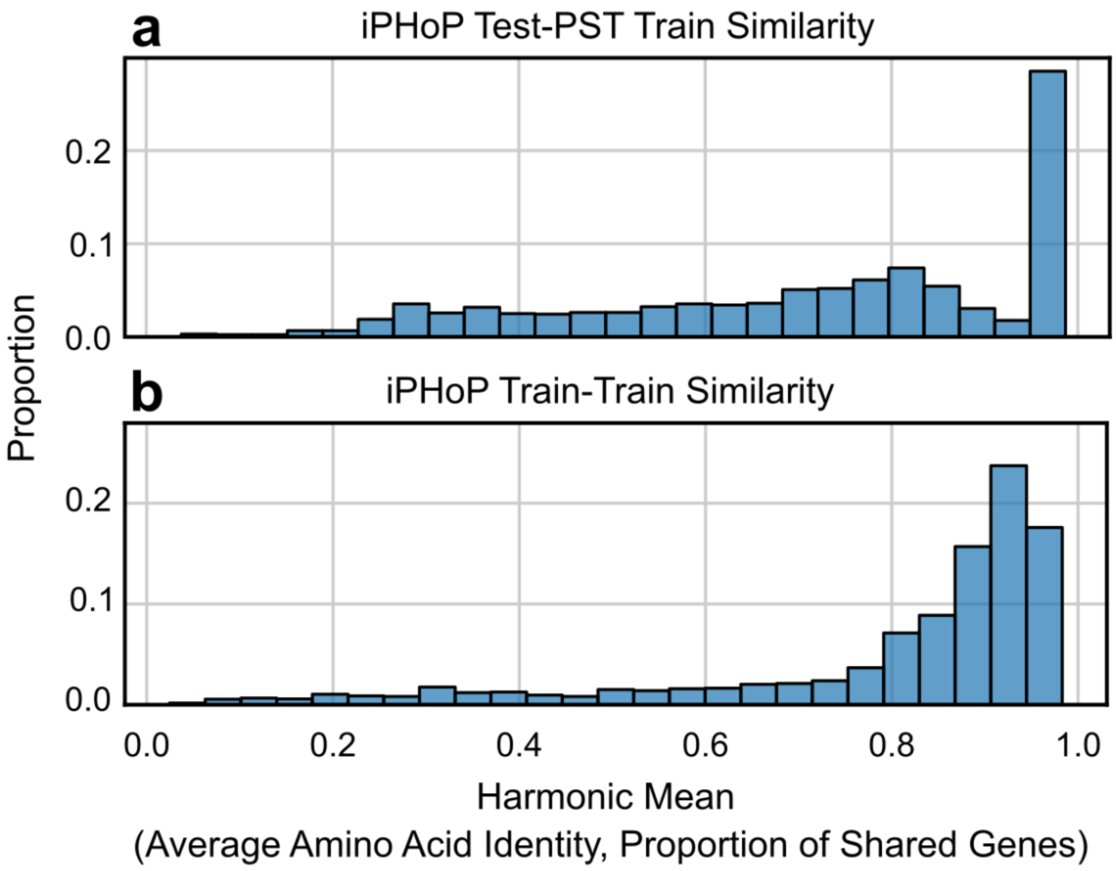

Supplementary Figure 25. iPHoP genome similarity evaluation. Genome-genome similarity between viruses from the indicated datasets. Genome-genome similarity was computed as the harmonic mean of the Average Amino Acid Identity and the proportion of shared genes between each pair of genomes. Then, the maximum similarity score for each iPHoP test virus when searching against the PST training dataset (**a**) or same dataset (**b**) was kept.

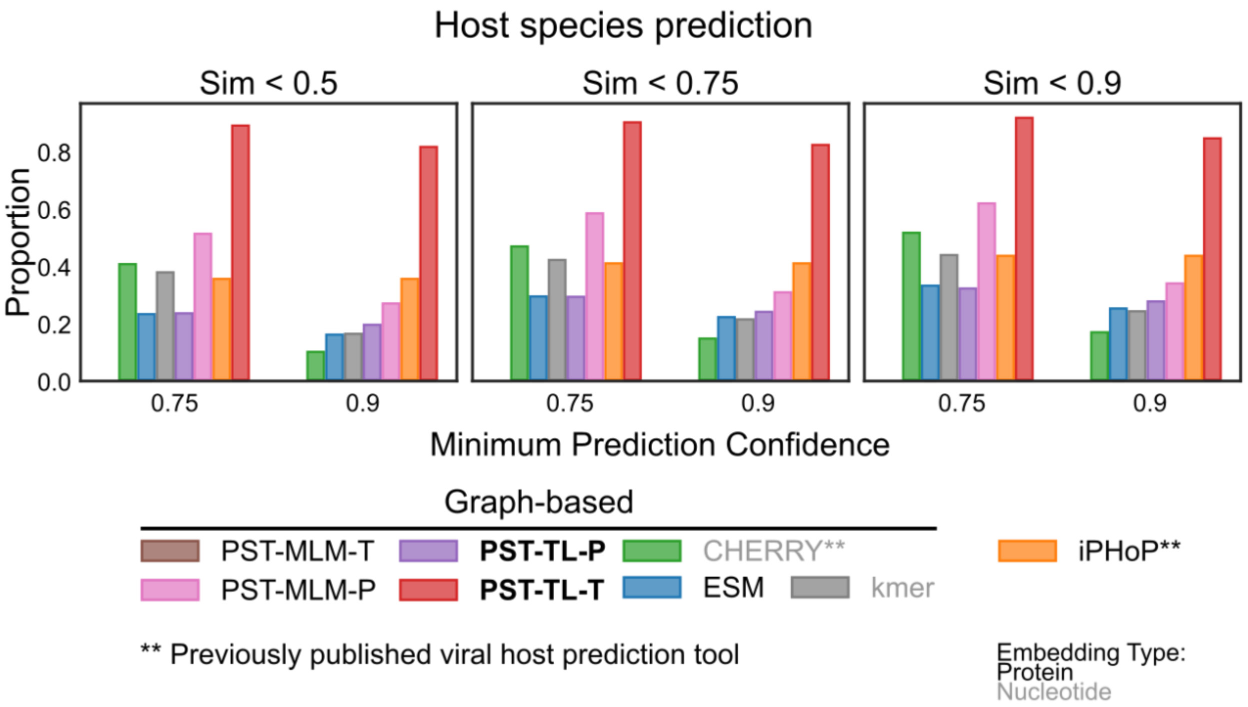

Supplementary Figure 26. Host prediction test set similarity to PST training set does not impact performance. Host species prediction performance removing viruses that were similar to the PST training dataset. Columns indicate the similarity threshold based on the harmonic mean of the Average Amino Acid Identity and the proportion of shared genes between each pair of genomes when comparing the iPHoP test and PST training datasets. Colors indicate the genome embedding type used to represent both the virus and host genomes in the interaction graph. The x-axis indicates the minimum prediction confidence required, and PST-MLM-T had no predictions above these thresholds.

SUPPLEMENTARY TABLES

Supplementary Table 1. Hyperparameter ranges and values to be sampled from for tuning PST.

Hyperparameter name	Name in code	Description	PST__small values	PST__large values
Model complexity	complexity	Number of attention heads and encoder layers	(Heads, Layers): {(4, 5), (8, 10), (16, 15), (32, 20), (32, 30)}	(Heads, Layers): {(4, 5), (8, 10), (16, 15), (32, 20)}
Positional and strand embedding scale	embed_scale	Size of both the positional and strand embeddings relative to the input embedding	{1, 1/2, 1/4, 1/8}	{1, 1/2, 1/4, 1/8} ** Note: The larger complexity models {(16, 15), (32,30)} were not allowed to have positional and strand embeddings equal to the input embedding.
Dropout	dropout	Dropout frequency	Uniform range: [0.0, 0.5]	Uniform range: [0.0, 0.5]
Layer dropout	layer_dropout	Dropout frequency of entire encoder layers	Uniform range: [0.0, 0.2]	Uniform range: [0.0, 0.2]
Lr	lr	Learning rate	Log uniform range: [5e-4, 5e-2]	Log uniform range: [5e-4, 5e-2]
Weight decay	weight_decay	Weight decaying regularizing parameter for AdamW optimizer	Log uniform range: [1e-3, 1e-1]	Log uniform range: [1e-3, 1e-1]
PST chunk size	chunk_size	Size (in number of proteins) of each genome subgraph	{15, 20, 25, 30, 35, 40, 45, 50}	{15, 20, 25, 30, 35, 40, 45, 50}
PointSwap rate	sample_rate	Rate of protein swapping during PointSwap data augmentation	Uniform range: [0.25, 0.75]	Uniform range: [0.25, 0.75]
Triplet loss scale	sample_scale	Negative exponential decay scale factor for negative example weight	Uniform range: [1.0, 15.0]	Uniform range: [1.0, 15.0]
Triplet loss margin	margin	How much farther away negative genome needs to be from the anchor than the positive. Positive genomes that are closer than the negative below this distance do not contribute to the loss.	Log uniform range: [0.1, 1.0]	Log uniform range: [0.1, 1.0]
Batch size	batch_size	Number of genomes per batch. Each batch is notably a different size due to a different number of proteins.	{8, 16, 32}	{8, 16, 32} ** Note: The larger complexity models {(16, 15), (32,30)} were not allowed to have the larger batch sizes due to memory.

Supplementary Table 2. Chosen hyperparameters for final PST models.

Hyperparameter name	PST-TL-T__small	PST-TL-T__large	PST-TL-P__small	PST-TL-P__large	PST-MLM-T__small	PST-MLM-T__large	PST-MLM-P__small	PST-MLM-P__large
Model complexity (Heads, Layers):	(4, 5)	(32, 20)	(4, 5)	(4, 5)	(4, 5)	(4, 5)	(32, 30)	(8, 10)
Positional and strand embedding scale	1/8	1/2	1/8	1/8	1	1	1	1
Dropout	0.3602979091	0.3081442146	0.1668379348	0.03041236888	0.2701386752	0.07528544676	0.2710076408	0.01064799387
Layer dropout	0.1992834076	0.1791077549	0.1808218609	0.03377253395	0.03760548452	0.1267757519	0.09778477102	0.05411254416
Lr	0.001145215852	0.0007510297928	0.0006974763989	0.0005549269152	0.002390565365	0.001705590579	0.001346451976	0.01364059918
Weight decay	0.09820296108	0.02877930738	0.01680200679	0.0759760899	0.03956876123	0.001840807545	0.08560339835	0.02272168583
PST chunk size	30	40	30	25	30	40	15	35
PointSwap rate	0.3262556009	0.4549705438	0.6101405214	0.6236630822	NA	NA	NA	NA
Contrastive loss scale	5.034412797	4.746652547	7.237959025	14.03376758	9.180845073	2.979390165	12.4366579	12.42113679
Triplet loss margin	1.152767646	0.6677220096	0.3345870543	0.5097346202	NA	NA	NA	NA
Batch size	32	8	32	32	32	8	16	32
Masking rate	NA	NA	NA	NA	0.15	0.25	0.2	0.3

Supplementary Table 3. Hyperparameter tuning trial summary for pretrained PST models.

ESM2 Input	Model Name	Completed	Pruned	Failed
ESM__small	PST-TL-T__small	45	29	1
ESM__large	PST-TL-T__large	16	22	16
ESM__small	PST-TL-P__small	7	46	0
ESM__large	PST-TL-P__large	10	78	1
ESM__small	PST-MLM-T__small	13	12	0
ESM__large	PST-MLM-T__large	15	5	0
ESM__small	PST-MLM-P__small	15	10	0
ESM__large	PST-MLM-P__large	10	10	0

Supplementary Table 4. Training summary of final pretrained PST models.

Model Name	Trained Epochs	Batch Accumulation Size	Training Time (h)	Number of Parameters
PST-TL-T__small	50	100	10.3	5,391,200
PST-TL-T__large	15	50	33.7	177,907,840
PST-TL-P__small	20	100	3.9	5,391,200
PST-TL-P__large	14	25	4.3	21,342,400
PST-MLM-T__small	41	25	4.5	23,751,680
PST-MLM-T__large	46	50	10.3	93,583,360
PST-MLM-P__small	37	25	14.8	93,030,080
PST-MLM-P__large	34	25	8.3	185,848,960

Supplementary Table 5. Regex patterns to VOG profiles into broader functional categories.

<table>
<thead>
<tr><th>Category</th><th>Regex Pattern</th></tr>
</thead>
<tbody>
<tr><td>structural</td><td>(?i)head|capsid|tail|baseplate|fiber|sheath|collar|neck|spike|plate|tube|connector|polyprotein|scaffold|interna|coat|structur|inner|assembl|adsorp|attach|envelop|tape|virion</td></tr>
<tr><td>packaging</td><td>(?i)terminase|portal|packag</td></tr>
<tr><td>exit</td><td>(?i)lysis|holin|spanin|Rz|lysin|lysozyme|peptidoglyc|muram|Virion export|budd</td></tr>
<tr><td>integration</td><td>(?i)integra|transpos|excision</td></tr>
<tr><td>gene expression</td><td>(?i)DNA-directed RNA pol|sigma|tRNA|RNA[-]binding|mRNA|RNA[-](lig|2'-phospho|silenc|helic|mediat|splic)|RNAP|transcrip|transla</td></tr>
<tr><td>anti-host defense</td><td>(?i)methyl|restri|modif|CRISPR|RNA-guided DNA endonuclease|apoptos|rII[AB $]</td></tr>
<tr><td>replication</td><td>(?i)DNA|repl|RNA[-]dep|RNA[-]directed|polyprotein|nucl|d[ATCG]TP|thymid|Phosphoribosylformylglycinamidine|puri|pyrimid</td></tr>
<tr><td>unknown</td><td>(?i)hypoth|uncharacter|unclass|unident|unknown|DUF|REFSEQ protein</td></tr>
</tbody>
</table>

Supplementary Table 6. Hyperparameter values to be sampled from for host prediction models.

Hyperparameter name	Name in code	Description	Values
Number of encoder convolution layers	encoder_n_layers	Number encoder convolution layers	{1, 2, 3, 4, 5}
Decoder layer sizes	decoder_hidden_dims	Dimensions of the 2-layer decoder MLP. Given then we tested a variety of embeddings of different sizes, values above the input embedding dimension were excluded.	{(512, 256), (512, 128), (512, 64), (512, 32), (256, 128), (256, 64), (256, 32), (128, 64), (128, 32), (64, 32)}
Learning rate	lr	AdamW optimizer learning rate	{5e-4, 1e-3, 5e-3}
Train edge supervision split	disjoint_train_ratio	Ratio of training edges used for supervision, leaving the remaining training edges for message passing. In the case of 0.0, all training edges are used for both.	{0.0, 0.3}
Share edge-type weights	share_weights	Whether to share learnable weights for each edge type, or to model edge-type-specific weights	{0, 1}

SUPPLEMENTARY DATA DESCRIPTIONS

Supplementary Data 1. Summary of PST training and test viral genomes.

Column number	Column	Description
1	genome_id	Unique genome ID per dataset. These correspond to the order of viral proteins (which are sorted per scaffold/genome) in the FASTA file used for obtaining ESM2 embeddings.
2	genome	Genome name in FASTA file. For multi-scaffold viruses in IMG/VR v4, this is the first field of each scaffold before a "|" separator.
3	dataset	Dataset descriptor. Possibly values: PST-TRAIN, IMGVRv4-TEST, MGnify-TEST
4	genome_length_bp	Length of genome in basepairs. For multi-scaffold viruses, this is the sum from each scaffold.
5	num_proteins	Number of predicted proteins using prodigal
6	scaffolds	Names of scaffolds that constitute each genome. For single-scaffold viruses, this is identical to the "genome" column. For multi-scaffold viruses from IMG/VR v4, each scaffold is separated by a ";".
7	taxonomy	GTDB style viral taxonomy string.
8	taxonomy_method	Method used to assign viral taxonomy. "geNomad" indicates using geNomad to assign taxonomy in this study. Other values indicate a different method or study used by the source database. NULL indicates the inability to assign a taxonomy.
9	viral_realm	Viral realm, if any
10	viral_kingdom	Viral kingdom, if any
11	viral_phylum	Viral phylum, if any
12	viral_class	Viral class, if any
13	viral_order	Viral order, if any
14	viral_family	Viral family, if any
15	viral_genus	Viral genus, if any
16	viral_species	Viral species, if any
17	completeness	Genome completeness as determined by CheckV if it could be determined
18	miuvig_quality	MIUViG[1] quality if it could be determined. For all genomes with a genome completeness value, this should be "High-quality"; otherwise, NULL.
19	sequence_origin	Source database with DOI accession of each viral genome. For sources that are composite databases like IMG/VR, more direct sources are also provided.
20	ecosystem_classification	GOLD[2] ecosystem classification. Most values are provided by source databases, but for single ecosystem databases, these were manually entered.
21	host_prediction_method	Method used for host prediction. For the training and IMG/VR v4 test viruses, these are provided by the source databases. For the MGnify test viruses, these were predicted by iPHoP in this study.
22	host_domain	Predicted host domain, if any
23	host_phylum	Predicted host phylum, if any
24	host_class	Predicted host class, if any
25	host_order	Predicted host order, if any
26	host_family	Predicted host family, if any
27	host_genus	Predicted host genus, if any
28	host_species	Predicted host species, if any
29	taxonomy_cv_group	Viral realm category used for taxonomic cross validation and loss weighting when used during training. For test viruses, this is NULL.
30	protein_div_cv_group	Viral protein diversity group used for protein diversity based cross validation. For test viruses, this is NULL.

Supplementary Data 2. Summary of predicted proteins from PST training and test viruses.

Column number	Column	Description
1	ptn	Protein name. All names follow prodigal format: "scaffold_number"
2	ptn_id	Unique protein ID per dataset. These correspond to the order of viral proteins (which are sorted per scaffold/genome) in the FASTA file used for obtaining ESM2 embeddings.
3	genome	Genome name in FASTA file. For multi-scaffold viruses in IMG/VR v4, this is the first field of each scaffold before a "|" separator.
4	genome_id	Unique genome ID per dataset. These correspond to the order of viral proteins (which are sorted per scaffold/genome) in the FASTA file used for obtaining ESM2 embeddings. This is the same ID found in Supplementary Data 1.
5	vog_bitscore	Bitscore to VOG profile, if any hits detected
6	vog_annot	Annotation of VOG profile, if any hits detected
7	vog_category	VOG category defined with regex patterns (Supplementary Table 5) searching annotation descriptions. Summary of categories for each VOG profile found in Supplementary Data 4.
8	phrog_bitscore	Bitscore to PHROG profile, if any hits detected
9	phrog_annot	Annotation of PHROG profile, if any hits detected
10	phrog_category	Curated PHROG functional category. See Supplementary Data 3 for descriptions.
11	dataset	Dataset descriptor of the genomes encoding these proteins. Possibly values: PST-TRAIN, IMGVRv4-TEST, MGnify-TEST

Supplementary Data 3. Curated PHROG protein annotation profiles.

Column number	Column	Description
1	phrog_id	PHROG v4 profile ID
2	phrog	Name of profile. All names are "phrog_id".
3	annot	Specific description of the function represented by the profile.
4	old_category	Functional category reported in the original PHROG v4 database.
5	new_category	Curated functional category used for this study.
6	changed_category	Whether or not new_category differs from old_category.

Supplementary Data 4. Broad functional categories for VOG r219 HMM profiles. See Supplementary Table 5 for patterns used to define the categories.

Column number	Column	Description
1	hmm	VOG HMM profile identifier.
2	n_ptns	Number of proteins used to construct profile (reported by VOG).
3	n_species	Number of species used in constructing profile (reported by VOG).
4	category	Original VOG functional category codes.
5	description	Specific description of the function represented by the profile.
6	function	Functional category used by this study. See Supplementary Table 5 for the regex patterns used to assign these labels based on the function description.

Supplementary Data 5. Viral-host prediction dataset summary.

Column number	Column	Description
1	dataset	Dataset descriptor (“train”, “test”) for each virus.
2	virus_accession	NCBI genome accession for virus.
3	virus_species	Virus species name.
4	host_accession	NCBI genome accession for host.
5	host_species	Full host species name provided by NCBI.
6	host_label	Cleaned version of host species name. Primarily removes information related to strain, serovar, etc.
7	gtdbtk_classification	Host taxonomic classification using GTDB r214. For hosts without classifications at the more specific ranks, a rank-specific identifier was appended to differentiate these from known hosts at the same rank.
8	n_viruses	Number of viruses infecting the indicated host.

SUPPLEMENTARY REFERENCES

1. Roux, S. *et al*. Minimum Information about an Uncultivated Virus Genome (MIUViG). *Nat Biotechnol* **37**, 29–37 (2018).

2. Ivanova, N. *et al*. A call for standardized classification of metagenome projects. *Environ Microbiol* **12**, 1803–5 (2010).
