# Supplementary material for: Protein Set Transformer: a protein-based genome language model to power high-diversity viromics
Source: Nat Commun. 2025 Nov 23;16:11123. doi: 10.1038/s41467-025-66049-4 (PMC12705698; doi:10.1038/s41467-025-66049-4)
Supplement: Supplementary file 2 — Reporting Summary [file 41467_2025_66049_MOESM2_ESM.pdf]

nature portfolio

Corresponding author(s): Karthik Anantharaman

Last updated by author(s): Sep 26, 2025

Reporting Summary

Nature Portfolio wishes to improve the reproducibility of the work that we publish. This form provides structure for consistency and transparency in reporting. For further information on Nature Portfolio policies, see our Editorial Policies and the Editorial Policy Checklist.

Statistics

For all statistical analyses, confirm that the following items are present in the figure legend, table legend, main text, or Methods section.

n/a	Confirmed	
☐	☒	The exact sample size (*n*) for each experimental group/condition, given as a discrete number and unit of measurement
☒	☐	A statement on whether measurements were taken from distinct samples or whether the same sample was measured repeatedly
☒	☐	The statistical test(s) used AND whether they are one- or two-sided<br>*Only common tests should be described solely by name; describe more complex techniques in the Methods section.*
☒	☐	A description of all covariates tested
☒	☐	A description of any assumptions or corrections, such as tests of normality and adjustment for multiple comparisons
☐	☒	A full description of the statistical parameters including central tendency (e.g. means) or other basic estimates (e.g. regression coefficient) AND variation (e.g. standard deviation) or associated estimates of uncertainty (e.g. confidence intervals)
☒	☐	For null hypothesis testing, the test statistic (e.g. *F*, *t*, *r*) with confidence intervals, effect sizes, degrees of freedom and *P* value noted<br>*Give P values as exact values whenever suitable.*
☒	☐	For Bayesian analysis, information on the choice of priors and Markov chain Monte Carlo settings
☒	☐	For hierarchical and complex designs, identification of the appropriate level for tests and full reporting of outcomes
☐	☒	Estimates of effect sizes (e.g. Cohen's *d*, Pearson's *r*), indicating how they were calculated

Our web collection on statistics for biologists contains articles on many of the points above.

Software and code

Policy information about availability of computer code

Data collection	No software was used to acquire the data for this study beyond standard Unix command line tools.
Data analysis	Standalone software: PhageBoost (v0.1.7), CheckV (v1.0.1), skani (v0.1.0), mcl (v14-137), geNomad (v1.7.4), mmseqs (v13.45111), iPHoP (v1.3.3)<br>Python libraries: pyrodigal (v2.3.0), pyrodigal-gv (v2.11.0), PyTorch (v2.1.0 & v2.2.2), fair-esm (v2.0.0), PyTorch-Geometric (v2.3.1 & v2.5.2), PyTorch-Lightning (v2.0.7 & v2.2.4), Optuna (v3.3.0), scikit-learn (v1.4.2), bionumpy (v1.0.8), faiss (v1.8.0), iGraph (v0.11.3), polars (v1.24), foldseek (v284d732b9a801c642213a396286cae3344a11d2c), pyhmmer (v.0.9.0), SciPy (v1.13.0)<br>Other ML models: Hyena-DNA (large-1m), GenSLM (genslm_25M_patric)<br>Code generated in this study is publicly available at https://github.com/AnantharamanLab/protein_set_transformer

For manuscripts utilizing custom algorithms or software that are central to the research but not yet described in published literature, software must be made available to editors and reviewers. We strongly encourage code deposition in a community repository (e.g. GitHub). See the Nature Portfolio guidelines for submitting code & software for further information.

Data

Policy information about availability of data

All manuscripts must include a data availability statement. This statement should provide the following information, where applicable:

- Accession codes, unique identifiers, or web links for publicly available datasets
- A description of any restrictions on data availability
- For clinical datasets or third party data, please ensure that the statement adheres to our policy

Sources for publicly available viral genomes are listed in Supplementary Data 1. All data specific to this manuscript, including protein FASTA files, protein and genome embeddings, trained PST model weights, virus-host interaction graphs, Supplementary Tables, Supplementary Data, and Source Data used for figure making, were deposited at DRYAD: (doi: 10.5061/dryad.d7wm37q8w). Data can be downloaded via the DRYAD web interface, and we have also provided command line access to download files with instructions in the PST GitHub repository. Descriptions of the Supplementary Data can be found in the Supplementary Information file, and all other necessary information is in the DRYAD README. The protein embeddings from PST-MLM models are not provided due to storage limitations in the DRYAD repository.

Research involving human participants, their data, or biological material

Policy information about studies with human participants or human data. See also policy information about sex, gender (identity/presentation), and sexual orientation and race, ethnicity and racism.

Reporting on sex and gender	N/A
Reporting on race, ethnicity, or other socially relevant groupings	N/A
Population characteristics	N/A
Recruitment	N/A
Ethics oversight	N/A

Note that full information on the approval of the study protocol must also be provided in the manuscript.

Field-specific reporting

Please select the one below that is the best fit for your research. If you are not sure, read the appropriate sections before making your selection.

☐ Life sciences ☐ Behavioural & social sciences ☒ Ecological, evolutionary & environmental sciences

For a reference copy of the document with all sections, see nature.com/documents/nr-reporting-summary-flat.pdf

Ecological, evolutionary & environmental sciences study design

All studies must disclose on these points even when the disclosure is negative.

Study description

Developed machine learning models trained on publicly available viral genomic data.

Research sample

Viral genome databases for training and evaluation:
1. Roux, S. et al. Ecogenomics and potential biogeochemical impacts of globally abundant ocean viruses. Nature 537, 689–693 (2016).
2. Tisza, M. J. & Buck, C. B. A catalog of tens of thousands of viruses from human metagenomes reveals hidden associations with chronic diseases. Proceedings of the National Academy of Sciences 118, e2023202118 (2021).
3. Cook, R. et al. Hybrid assembly of an agricultural slurry virome reveals a diverse and stable community with the potential to alter the metabolism and virulence of veterinary pathogens. Microbiome 9, 65 (2021).
4. Gazitúa, M. C. et al. Potential virus-mediated nitrogen cycling in oxygen-depleted oceanic waters. ISME J 15, 981–998 (2021).
5. Gregory, A. C. et al. Marine DNA Viral Macro- and Microdiversity from Pole to Pole. Cell 177, 1109-1123.e14 (2019).
6. Parks, D. H. et al. GTDB: an ongoing census of bacterial and archaeal diversity through a phylogenetically consistent, rank normalized and complete genome-based taxonomy. Nucleic Acids Research 50, D785–D794 (2022).
7. Camarillo-Guerrero, L. F., Almeida, A., Rangel-Pineros, G., Finn, R. D. & Lawley, T. D. Massive expansion of human gut bacteriophage diversity. Cell 184, 1098-1109.e9 (2021).
8. Gregory, A. C. et al. The Gut Virome Database Reveals Age-Dependent Patterns of Virome Diversity in the Human Gut. Cell Host Microbe 28, 724-740.e8 (2020).
9. Roux, S. et al. IMG/VR v3: an integrated ecological and evolutionary framework for interrogating genomes of uncultivated viruses. Nucleic Acids Research 49, D764–D775 (2021).
10. Michniewski, S. et al. A new family of “megaphages” abundant in the marine environment. ISME COMMUN. 1, 1–4 (2021).
11. ter Horst, A. M. et al. Minnesota peat viromes reveal terrestrial and aquatic niche partitioning for local and global viral populations. Microbiome 9, 233 (2021).
12. Brum, J. R. et al. Patterns and ecological drivers of ocean viral communities.

Science 348, 1261498 (2015).
13. Camargo, A. P. et al. IMG/VR v4: an expanded database of uncultivated virus genomes within a framework of extensive functional, taxonomic, and ecological metadata. Nucleic Acids Res 51, D733–D743 (2023).
14. Richardson, L. et al. MGnify: the microbiome sequence data analysis resource in 2023. Nucleic Acids Research 51, D753–D759 (2023).

Sampling strategy	N/A
Data collection	We used publicly available viral genomes.
Timing and spatial scale	N/A
Data exclusions	N/A
Reproducibility	N/A
Randomization	N/A
Blinding	N/A

Did the study involve field work? ☐ Yes ☒ No

Reporting for specific materials, systems and methods

We require information from authors about some types of materials, experimental systems and methods used in many studies. Here, indicate whether each material, system or method listed is relevant to your study. If you are not sure if a list item applies to your research, read the appropriate section before selecting a response.

Materials & experimental systems

n/a	Involved in the study	
☒	☐	Antibodies
☒	☐	Eukaryotic cell lines
☒	☐	Palaeontology and archaeology
☒	☐	Animals and other organisms
☒	☐	Clinical data
☐	☒	Dual use research of concern
☒	☐	Plants

Methods

n/a	Involved in the study	
☒	☐	ChIP-seq
☒	☐	Flow cytometry
☒	☐	MRI-based neuroimaging

Dual use research of concern

Policy information about dual use research of concern

Hazards

Could the accidental, deliberate or reckless misuse of agents or technologies generated in the work, or the application of information presented in the manuscript, pose a threat to:

No	Yes	
☐	☒	Public health
☐	☒	National security
☐	☒	Crops and/or livestock
☐	☒	Ecosystems
☒	☐	Any other significant area

Hazards	We have trained a large machine learning model exclusively on viral genomes. While there are no concrete examples that directly link our work to these potential hazards, one could argue that our work may increase the ability to guide the generation of more pathogenic variants of viruses that could affect these areas. We conducted both internal and an independent external biosecurity risk assessment that both agreed that the potential risk was minimal.

For examples of agents subject to oversight, see the United States Government Policy for Institutional Oversight of Life Sciences Dual Use Research of Concern.

Experiments of concern

Does the work involve any of these experiments of concern:

No	Yes	
☒	☐	Demonstrate how to render a vaccine ineffective
☒	☐	Confer resistance to therapeutically useful antibiotics or antiviral agents
☒	☐	Enhance the virulence of a pathogen or render a nonpathogen virulent
☒	☐	Increase transmissibility of a pathogen
☒	☐	Alter the host range of a pathogen
☒	☐	Enable evasion of diagnostic/detection modalities
☒	☐	Enable the weaponization of a biological agent or toxin
☒	☐	Any other potentially harmful combination of experiments and agents

Precautions and benefits

Biosecurity precautions	Our machine learning model only operates in an abstract linear algebra embedding space, in which it is not trivial to reverse translate back into specific protein and genome sequences. This significantly limits generation of de novo genomes from our model for the creation of designer viral genomes.
Biosecurity oversight	We conducted an independent biosecurity review of our work with experts in computational virology and machine learning.
Benefits	The potential to increase our understanding of viruses and their host interactions brings basic and, subsequently, applied outcomes. For example, while the major concern is the generation of more pathogenic viruses, a counterargument is the generation of viruses that better control pathogenic microbial populations that in turn benefits humans and crops/livestock.
Communication benefits	Our work aims to increase our understanding of viruses, of which the vast majority in the world infect bacteria and likely are unable to pose significant direct or indirect threat to humans. Communicating our work has implications for understanding significant contributors to ecological communities. Additionally, the methodology developed here may also be useful for better understanding microbial protein function.

Plants

Seed stocks	N/A - this field won't leave
Novel plant genotypes	N/A - this field won't leave
Authentication	N/A - this field won't leave
